# Free Fatty Acid Receptors 2 and 3 as Microbial Metabolite Sensors to Shape Host Health: Pharmacophysiological View

**DOI:** 10.3390/biomedicines8060154

**Published:** 2020-06-08

**Authors:** Sidharth P. Mishra, Prashantha Karunakar, Subhash Taraphder, Hariom Yadav

**Affiliations:** 1 Department of Internal Medicine, Molecular Medicine, Wake Forest School of Medicine, Winston-Salem, NC 27101, USA; spmishra@wakehealth.edu; 2 Department of Animal Genetics and Breeding, West Bengal University of Animal and Fishery Science, Kolkata, West-Bengal 700037, India; subhash.taraphder@gmail.com; 3 Department of Biotechnology, PES University, Bangalore, Karnataka 560085, India; prashanthakarunakar@pes.edu; 4 Department of Microbiology and Immunology, Molecular Medicine, Wake Forest School of Medicine, Winston-Salem, NC 27101, USA

**Keywords:** FFAR2, FFAR3, microbiota, gut, immune, SCFA

## Abstract

The role of the gut microbiome in human health is becoming apparent. The major functional impact of the gut microbiome is transmitted through the microbial metabolites that are produced in the gut and interact with host cells either in the local gut environment or are absorbed into circulation to impact distant cells/organs. Short-chain fatty acids (SCFAs) are the major microbial metabolites that are produced in the gut through the fermentation of non-digestible fibers. SCFAs are known to function through various mechanisms, however, their signaling through free fatty acid receptors 2 and 3 (FFAR2/3; type of G-coupled protein receptors) is a new therapeutic approach. FFAR2/3 are widely expressed in diverse cell types in human and mice, and function as sensors of SCFAs to change several physiological and cellular functions. FFAR2/3 modulate neurological signaling, energy metabolism, intestinal cellular homeostasis, immune response, and hormone synthesis. FFAR2/3 function through Gi and/or Gq signaling, that is mediated through specific structural features of SCFAs-FFAR2/3 bindings and modulating specific signaling pathway. In this review, we discuss the wide-spread expression and structural homologies between human and mice FFAR2/3, and their role in different human health conditions. This information can unlock opportunities to weigh the potential of FFAR2/3 as a drug target to prevent human diseases.

## 1. Introduction

The gut microbiome and its contribution to human health is an emerging area and much remains to be learned about the interactions between microbial cells with a host. The major communication between the microbiome and host cells takes place through the metabolites produced by the gut microbiome [1]. These metabolites are sensed by host cells through various mechanisms. The major class of microbial metabolites are short-chain fatty acids (SCFAs; such as acetate, propionate, and butyrate) that are either utilized by intestinal cells and/or are absorbed and enter circulation [2,3,4,5,6,7,8,9]. One of the major pathways by which SCFAs function on target cells is by activating free fatty acid receptors 2 and 3 (FFAR2/3), that are types of G-coupled protein receptors (GPCRs) [3,4,5]. FFAR2/3 are abundantly expressed on intestinal cells and other cell types in the host and known to regulate various physiological and cellular functions. Both the receptors are differentially expressed in the intestine [3,10,11,12], adipose tissues [13,14], pancreas [15], bone marrow [10], liver [16,17], muscle [18,19], spleen [20,21], lungs [6,21,22], heart [23], and brain [17,24]. Widespread expression of FFAR2/3 make them play an important role in several human diseases such as type 1 and type 2 diabetes [11,25,26,27,28,29], obesity [26,30,31,32,33], inflammatory bowel disease (IBD) [34,35], Crohn’s disease [36], cardiovascular diseases [20,37,38], gout [39], asthma [6,40,41], arthritis [35], and colitis [35,42,43,44,45].

FFAR2/3 are cell surface receptors that can play a very significant role in intracellular cell signaling [46,47]. Both FFAR2/3 receptors activate the heterotrimeric G-coupled protein intracellularly, by binding with endogenous SCFAs at the cell surface of any specific tissues. Both FFAR2 and FFAR3 are characterized as seven transmembrane (7TM) spanning proteins and consist of ~2% of the whole human and mice genome sequence respectively [46], and are coupled with Gα_i/o_ coupled signaling [22,48]. Only FFAR2 is associated with Gα_q/11_ coupled signaling [32,49]. Therefore, based on expression pattern, structural importance, and activation of FFAR2/3 receptors by gut microbiota metabolites at different tissue levels provide a new scope to investigate the importance of these receptors on human diseases. However, sources of information about detailed expression patterns, structural and functional analyses, and their biological functions in different human diseases and health conditions is obscure.

## 2. Experimental Section

Here we performed an extensive search of the literature and compiled detailed information about the expression (gene and protein) patterns and comparative structural analysis developed by using our own in-silico designed model setup of the FFAR2/3 receptors. We described the expression information from literature as well as from our cell line repository. In addition, we have performed a literature search using PubMed, Google Scholar, and Web of Knowledge using different combinations of keywords FFAR2, FFAR3, GPR43, GPR41, microbiota, short-chain fatty acids, diet, fibers, human health, diabetes, obesity, colon, intestine, adipose tissue, liver, lungs, disease, metabolic disease, stem cell, monocyte, Colitis, expression, structure, modeling, mice, knockout, human, agonist, antagonist, activator, inhibitor, software, cell line, enteroendocrine, ligand, docking, tight junction, inflammation, mucus, brain, neuron, and organoid with diverse combinations.

Protein sequences and structural information were obtained from the protein database site and modeled by using modeler software and I-TASSER to develop the models for further docking with ligands, and also comparing and verifying with specific ligands in human and mice. More comprehensive information about FFAR2/3 in-silico-interactive analysis have also been published elsewhere [50,51,52]. This article will provide one stop, detailed, and the most current information about the FFAR2/3 expression, structure, and their biological roles.

### 2.1. Expression of FFAR2 and FFAR3 in Different Species and Tissues/Cells

**Figure 1:**
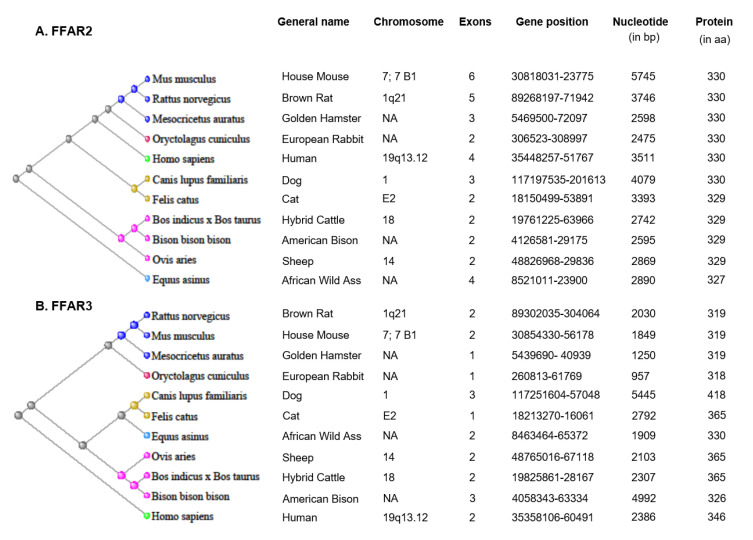
Phylogenetic tree depicting genetic closeness and differences in Free fatty acid receptor 2 (FFAR2) (**A**) and Free fatty acid receptor 3 (FFAR3) (**B**) among different animal species.

FFAR2 and FFAR3 are present in close proximity on chromosome 1 (19q13.12) in humans and chromosome 7 (7; 7 B1) in mice. The phylogenetic tree and detail information on FFAR2/3 chromosomal location, gene length, and position, number of exons, and protein length are shown in Figure 1. FFAR2/3 are widely expressed in different human and mice tissues, cells and cell lines, and in other species too. Below, we summarized the updated information about the expression of FFAR2/3 in humans, rodents, and other species, along with their cell types.

#### 2.1.1. FFAR2 Expression

During the initial days of FFAR2 discovery, it was found to be intensively expressed in human immune cells such as peripheral blood mononuclear cells (PBMCs) and polymorphonuclear cells (PMNs) with maximum expression in neutrophils [53,54,55,56,57]. However, more recent studies and our own data show that FFAR2 is expressed in human fungiform taste buds [58], dendritic cells (DCs) derived from bone marrow [59], liver [55], heart [55], pancreatic islet of Langerhans [60,61], spleen [55], fetal membranes and placenta [62], L-cells in the large intestine [35,63], brain parenchyma [64], neuronal cell line- SK-N-SH, and the human breast cancer cell line (MCF-7) [65]. Additionally, FFAR2 expressed in colonic epithelia and mucosa but not in the colonic muscle and submucosal regions [66]. Its expression in muscle remains controversial [55,67]. Although FFAR2 expression is seen in brain parenchyma, comprehensive studies on its expression in the brain are needed to define its importance in brain functions [64].

FFAR2 expresses in hypothalamus [19], bone marrow [10,59], heart [68], liver [4,5,19], PBMC [35,53,54,55,56,69], stomach [19], pancreatic islet β-cells [25], lungs [40], epidermal fat pads [4,19], white adipose tissue (WAT) including perirenal, epidermal, and subcutaneous tissues [22,26], ileum and colon [4,19] particularly intestinal epithelial cells (IECs) [28], L-cells [11], I-cells [3], K-cells [3] and Myeloid (M)-cells [70,71], cecum [72], and muscles [19] of mice. FFAR2 also express in the murine pancreatic β-cell line MIN6 [60,61,73] and adipocyte cell line 3T3-L1 [19,30,31,74,75]. FFAR2 was found to be expressed in enteroendocrine cells and mucosal mast cells of rat and mice [35,76,77]. In both mice and rat, FFAR2 expresses only in the epithelial and mucosal layer but not in muscle and submucosal region of the colon [19,66,78]. Expression of FFAR2 in the central nervous system [77] and brown adipose tissue (BAT) [30,33] is unclear, and needs to be further studied.

FFAR2 expresses in the hypothalamus, cerebral cortex [79], pituitary gland [79], heart [79], liver [79,80], pancreas [79], rumen [79] particularly rumen papillae [81], omasum [79], reticulum [79], spleen [79], kidney [79], adrenal gland [79], colon [79], adipose tissues [82,83], and skeletal muscle [79,83] of bovines. FFAR2 expression on bovine adipose tissue and testis is still debatable [12,79,84]. It also expresses in the bovine mammary epithelial cell line (bMEC) [85]. In swine, FFAR2 expresses in the heart [13], liver [13], spleen [13,86], pancreas [86], adipose tissues [13,86,87], kidney [13], small intestine [88], caecum [88], colon [88], and skeletal muscle [13]. In sheep, FFAR2 expresses in abomasal (distal gastric) lymph nodes [89]. In New Zealand rabbits FFAR2 expresses in thymus, spleen, pancreas, adipose tissue, lungs, duodenum, jejunum, cecum ileum and colon [90]. In chickens, FFAR2 paralog genes are expressed in the testis, spleen, peripheral blood mononuclear cells (PBMC)s, adipose tissues, intestine, lung, liver, pancreas, ovary, thigh muscle, pectoralis muscle, eye, skin, subcutaneous adipose tissues, kidney, brain, heart, and uropygial gland [91]. Feline and canine soft tissue sarcoma as well as normal skin of felines and skeletal muscle of canines also showed the FFAR2 expression [92]. FFAR2 is also found to be expressed in horse placenta [93]. However, FFAR2 expression on other experimental models is still not known.

#### 2.1.2. FFAR3 Expression

FFAR3 expression is detected in the central nervous system (CNS) [53], brain endothelium [64], sympathetic nervous system (SNS) [23,64,94], bone marrow [53,54], liver [67], spleen [53,54], pancreatic β-cells [95,96], WAT [53,54,97], fetal membrane along with the mother placenta [62], small and large intestine [53,67], skeletal muscles [67], lymph node [53], and immune cells [41,53,54] of humans. It is also expressed in human breast cancerous cell lines such as MCF-7 [98], colorectal cancer (CRC) cell line HCT116 [99] and human embryonic kidney cell line HEK293 [100].

In rodents, FFAR3 expression is detected in superior cervical ganglia (SCG) and celiac sympathetic-mesenteric ganglia (CSMG) of autonomic nervous systems selected randomly from different tissue regions [23,94,101,102], peripheral nervous system (PNSs) such as the enteric nervous system and sensory neurons [3,23,103,104], the hypothalamic region of brain [19], heart [68], liver [5,19], stomach [19], pancreatic β-cells [95,96], adipose tissues [97], intestine (ileum and colon) [19,67,105] in the IECs [28,106], tuft cells [107,108,109], neuropeptide precursors and neurotensin (NeurogD3 and Neurogenin3) enteroendocrine cells such as L-cells [11], I-cells [3], K-cells [3], enteric neurons [3], colonic mucosa [78], and skeletal muscles [67]. It is also expressed in mouse cell lines such as Hepa1-6 [19,67], 3T3-L1 [53,74,75,110], 3T3-F442A [53], Ob-Luc [97] and myotubes C2C12 [110].

FFAR3 expression is detected in bovine cerebral cortex [79], hypothalamus [79], pituitary gland [79], heart [79], lungs [79], liver [79], pancreas [79], spleen [79], rumen [79,81], kidney [79], adrenal gland [79], adipose tissue [12,82,84], duodenum [79], colon [79], skeletal muscle [79], and thw bMEC cell line [85]. In swine, FFAR3 expresses in the heart [13], kidney [13], liver [13], adipose tissue [86], spleen [13,86], gastrointestinal (GI) tract [88], and skeletal muscle [13]. However, FFAR3 expression in swine adipose tissues is debatable [13,86]. FFAR3 gene expression was found in the colon, cecum, thymus, spleen, pancreas, adipose tissue, lungs, duodenum, jejunum, and ileum of the New Zealand rabbit [90]. FFAR3 gene was reported to be absent in chicken genome [91]. FFAR3 was also found to express in adipose tissues of goats [111] and sheep [112].

**Figure 2:**
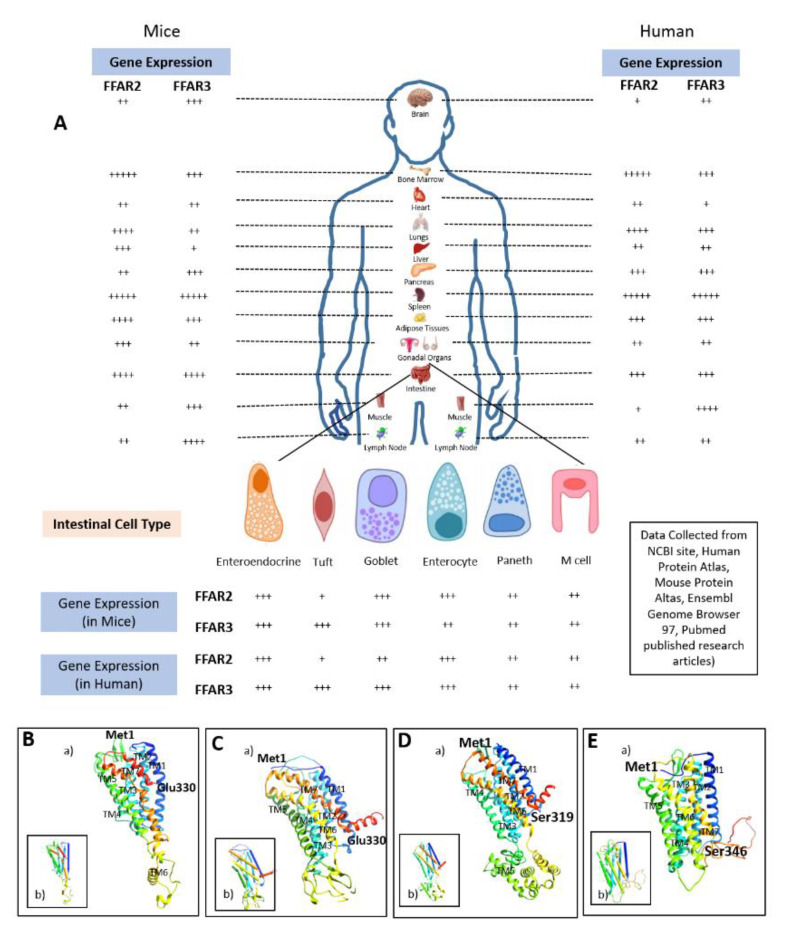
(**A**) Diagrammatic representation of FFAR2 and FFAR3 expression in human tissues/cells and their comparison with mouse tissues/cells. (**B**–**E**) Homolgy structure of mouse (**B**,**D**) and human (**C**,**E**) FFAR2 (**A**,**B**) and pFFAR3 (**D**,**E**) protein. (a,b) Depicts the rainbow (a) and pipes and plank (b) structures.

Comprehensive expression analysis of FFAR2 and FFAR3 in different tissues and cell lines of mice and humans are presented in Table 1 and Figure 2.

## 3. Structures of FFAR2 and FFAR3

Multiple emerging sources indicate that FFAR2/3 can be novel targets to prevent and/or treat several human diseases [2,39,60,113,114,115,116]. However, lack of knowledge in their structure and precise understanding of their interactions with ligands leads to a delay in gaining attention to be considered as novel therapeutic targets. However, growing understanding using evolved computational in-silico analyses and their significant role in several human diseases such as obesity, diabetes, IBD, and aging, FFAR2/3 are emerging as potential therapeutic targets [117,118]. Homology modeling of FFAR2 and FFAR3 along with gene mutagenesis, structural conformation, and protein-ligand interaction is developed and their importance is discussed in the following section.

### 3.1. FFAR2

The crystal structure of FFAR2 is not yet determined and the structure was predicted using human β_2_-adrenergic receptor as template. [50]. Human FFAR2 comprises 330 amino acids (AAs) that are arranged in a 7-TM structure [117,129]. Structurally, the third TM of FFAR2 contains cysteine residue at the top and an arginine residue at the bottom [100,118], and a conserved domain of GPCR family- Glu-Arg-Tyr motif [100]. The active site of human FFAR2 consists of Tyr^90^, Ile^145^, Arg^180^, Arg^255^, and Glu^166^ [46,52]. The small carboxylic acids (SCAs) bind to this binding pocket of Tyr^90^, Ile^145^, and Glu^166^ [52]. Arg^180^ and Arg^255^ are positively charged orthosteric sites which interact with negatively charged glutamine residue (Glu^171^) to stabilize the protein structure for proper binding with ligands [46].

**Figure 3:**
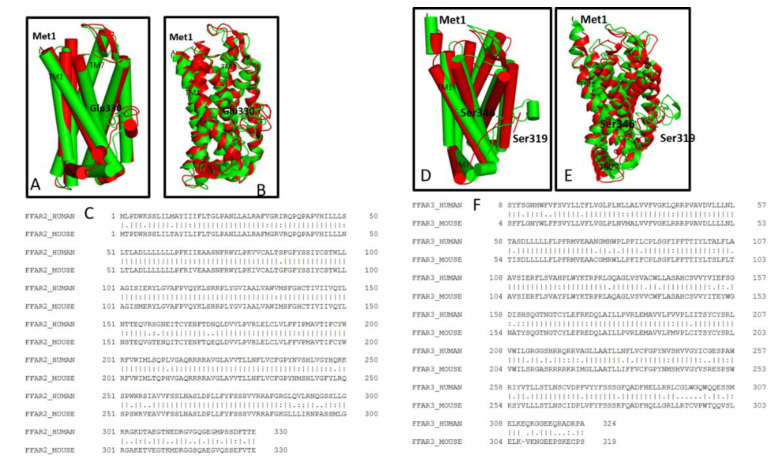
Superposition of mice and human FFAR2 and FFAR3 receptors (**A**–**F**) Pipes and Plank model (**A**,**D**); Rainbow Ribbon Model (**B**,**E**); and root-mean-square deviation (RMSD) pairwise alignments of mouse and human FFAR2 (**A**–**C**) and FFAR3 (**D**–**F**) receptor sequence.

Similar to humans, mouse FFAR2 is also a 7-TM protein made of 330 AAs [129] and shows 81.69% nucleotide level and 84.85% protein level similarity to it. The mouse FFAR2 receptor sequence superposition and pairwise alignment with human FFAR2 is shown in Figure 3A–C The active binding site of mice FFAR2 is comprised of Trp^75^, Gln^148^, Tyr^238^, Arg^65^, Arg^180^, Tyr^90^, and Arg^255^ according to our in-silico analysis. Both mouse and human FFAR2 show the protein sequential similarity from the 95-111 AAs position except at the 105^th^ position where mouse FFAR2 consists of methionine whereas human FFAR2 is of isoleucine. The significant change in the secondary structure of human and mice FFAR2 is observed at the C-terminal end. The C-terminal of the receptors consists of most conserved minimotifs and short peptides that regulate receptor binding efficiency, posttranslational modification, and trafficking with unique biochemical and physiological properties [130]. The detailed information on C- termini biophysical properties of FFAR2/3 are out of context and yet to be studied in a more comprehensive manner.

The FFAR2-like protein in chicken contains 367 AAs, encoded with the gene of 1105 bp nucleotides as per National Center for Biotechnology Information (NCBI)’s latest update. The chicken FFAR2 paralog homology model shows four active AA residues at the positions His^140^, Arg^180^, His^242^, and Arg^255^ [91]. These residues are further supported by Thr^201^, Glu^113^, and His^115^, and associated with protein-ligand interactions [91]. The homology models and chromosomal location of other laboratory animal species (*Mesocricetus auratus*, *Cavia porcellus*, and *Oryctolagus cuniculus*) are not yet known and studies are needed to comprehend their location, structure, and functionalities.

### 3.2. FFAR3

Human FFAR3 structure was also predicted based on the crystal structure of the human β_2_-adrenergic receptor [50]. Human FFAR3 is made of 346 AAs and has 52% of AA sequence similarity with mice FFAR2 [117,129]. Similar to FFAR2, FFAR3 also contains an arginine at the bottom of the third TM domain [100,118] which contains Glu-Arg-Phe motif of GPCR family class A [100]. AA residues Phe^96^, Tyr^151^, and Leu^171^ are involved in specific ligand binding [52] with SCAs including SCFAs. The presence of positively charged Leu^171^ residues provides stabilization to negatively charged arginine residues at the second extracellular loop (EL2) [46]. The olymorphism of FFAR3 can be considered for detailed comprehensive genetic and pharmacophysiological study against various diseases [94,131]. Mouse FFAR3 is 319 AAs long [129] and has similarity of 80.41% (nucleotide level) and 76.66% (protein level) with human FFAR3. Our, in-silico analysis between human and mouse FFAR3 proteins showed two substitutions at A103S and A107T site, and significant differences in human and mouse FFAR3 secondary structure was found at the C-terminal end. A superimposed structure and pairwise alignment of human and mice FFAR3 is shown in Figure 3D–F Detail information on chromosomal location and structural analyses of FFAR3 protein from other species are not available, and need further comprehensive studies.

### 3.3. Comparative Structural Analyses of FFAR2 and FFAR3

The structure-activity relationship (SAR) study showed that the endogenous binding site volume of human homology FFAR3 (105 Å^3^) is twice more than the volume of FFAR2 (41 Å^3^) [51]. SAR helps in determining the chemical structure of a receptor, its relationship with the chemical compounds associated with any biological activity, and chemical structural modification in the receptors to increase the biological activity of the compound [132]. Along with SAR, solvent accessible surface area (SASA) also helps in determining the molecular interaction of a biomolecule with the surrounded solvent to judge its biological effect on the organism [133]. SASA analysis of human and mice FFAR2/3 revealed that both have significantly higher hydrophobic residues than hydrophilic residues [52]. Human FFAR2 receptors have higher SASA hydrophobicity by 39 Å^2^ and higher aromatic SASA value by 63 Å^2^ as compared to human FFAR3 [52]. Based on virtual docking of different allosteric compounds to these receptors, it was found that FFAR2 pockets have larger volumes (553 Å^3^) and surface areas (510 Å^2^) as compared to FFAR3 binding pockets [117]. However, the volume of the FFAR3 binding cavity (385 Å^3^) is larger than FFAR2 (332 Å^3^) [52] whereas the depth of FFAR2 pockets is less than FFAR3 pockets by 2 Å [117]. Therefore, focus must be given to the compounds with diverse small-part SCAs (having lipophilic tails such as branched, cyclic, and unsaturated structures) for proper binding with FFAR2/3. According to Tikhonova et al. [52], prediction for the receptor subtype with preferably selective binding residues between FFAR2/3 are Tyr^90^, Ile^145^, and Glu^166^ in FFAR2 and Phe^96^, Tyr^151^, and Leu^171^. Thus, SAR information shows that FFAR2 and FFAR3 are lying in very close proximity to each other and can interact with the same chemical compound to compensate for each other’s biological response [99]. Thus, more intensive and precise study must be done to determine the individual biological function of each receptor and its binding to a particular ligand.

## 4. Interactions of SCFAs with FFAR2/3

### 4.1. FFAR2 Interaction with SCFA

**Figure 4:**
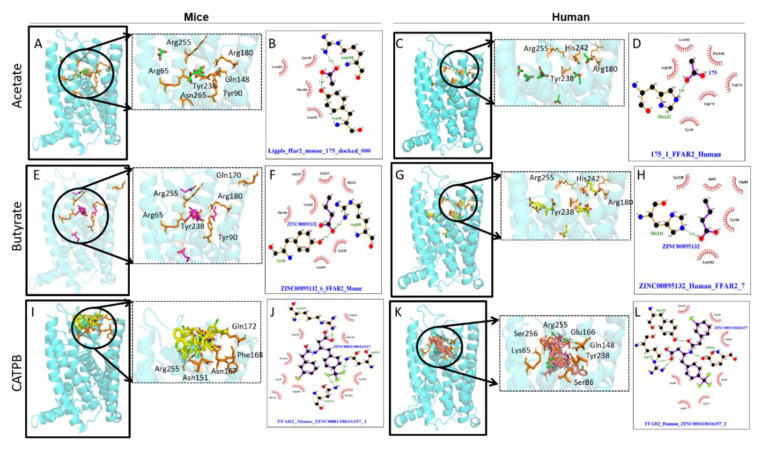
Structural analyses of FFAR2 protein-ligand bindings with agonists–acetate (**A**–**D**) and butyrate (**E**–**H**) and an FFAR2 antagonist-CATPB ((S)-3-(2-(3-chlorophenyl)acetamido)-4-(4-(trifluoromethyl)phenyl)butanoic acid) (**I**–**L**) in the ribbon models (**A**,**C**,**E**,**G**,**I**,**K**) and two dimensional Ligplot images (**B**,**D**,**F**,**H**,**J**,**K**)) and of mice (**A**,**B**,**E**,**F**,**I**,**J**) and human (**C**,**D**,**G**,**H**,**K**,**L**).

SCFAs are the orthosteric ligand of FFAR2/3 as they bind to endogenous binding sites [51]. SCFAs’ carboxylic group interacts with the arginine groups of third, fifth, and/or sixth TM domain of FFAR2 for efficient binding [51]. This SAR data explained that FFAR2 prefers flat, unsaturated moieties within the SCAs [51]. As a result, FFAR2 mostly binds to the ligands with sp^2^- or sp-hybridized α-carbon [51]. That means carbon atoms of SCAs form covalent bonds with either two or one hydrogen (H) atoms for interaction with FFAR2. This concept has been further justified by Tikhonova and Poerio [52] by showing that the FFAR2-selective binding with tiglic acid as an orthosteric ligand (binding of the ligand at the endogenous site) forms a network-intensive H-bond, while leaving a small binding cavity in FFAR2. However, substitutional mutation of histidine at His^140^ and His^242^ residues to alanine in the fourth and sixth TM domains decrease the binding potential of SCFAs to FFAR2 [128,134]. Using site-specific mutagenesis revealed that arginine (Arg^180^, Arg^255^) mutation at the top of either five and/or seven TM helix is important for facilitating the interactions of SCFAs with human and mice FFAR2 [100] (Figure 4). Through FFAR2 signaling, acetate moved to the peripheral tissue to regulate lipogenesis, cholesterol metabolism, and control central appetite [135]. Moreover, propionate is responsible for maintaining the whole body’s energy metabolism by controlling satiety signaling via FFAR2 [17,24]. Activated FFAR2 signaling by propionate treatment to human breast mesenchymal-like MDA-MB-231 and MDA-MB-436 cell inhibited the Hippo-Yap pathway to reduce metastatic [126]. In addition, FFAR2 signaling mediated by butyrate treatment to human enteroendocrine cell lines such as NCI-H716 (colorectal cell line) and HuTu-80 (duodenal cell line) increase Peptide YY (PYY) gut hormonal synthesis [125].

So far, no in-silico study demonstrated the SCFAs’ binding site with FFAR2 receptor in mice. Recently, we determined the common active binding site residues (*n* = 31) of mouse FFAR2 through homology modeling (unpublished data). The mouse FFAR2 interacts with SCFAs by forming H-bonds, such as acetate-made H-bonds with Tyr^90^, Gln^148^, Trp^75^, and Arg^65^; propionate-made H-bonds with Tyr^90^, Ile^145^, Arg^255^, Tyr^238^, Arg^180^, and His^242^; and butyrate-made H-bonds with Trp^75^, Gln^148^, Tyr^238^, Arg^65^, Arg^180^, Tyr^90^, and Arg^255^. Similar to humans, the mouse FFAR2 activated by acetate and butyrate by making H-bonds with Arg^180^, Tyr^190^, and His^242^ residual sites [26,125]. In mice as well, the activated FFAR2 receptor regulates biological functions such as hormonal synthesis [125], systemic inflammation [18,45], lipid metabolism [87], and adipogenesis [74,75] in maintaining body homeostasis. Detailed studies on the binding of SCFAs with FFAR2 of other species organisms are needed.

Therefore, in both humans and mice, SCFAs are associated with activation of FFAR2 in regulating biological functions such as incretin hormonal synthesis [24], metabolic syndrome [25,28,36], and occurrence of autoimmune diseases [20,136] in host. Therefore, these findings provide opportunities to study in detail which biological functions are regulated by FFAR2 and simultaneously screen the synthetic molecules for effective activation of FFAR2 for effective biological response by either inhibiting the mutation or changing the structural form of the receptor.

### 4.2. FFAR3 Interaction with SCFA

SAR data showed that human FFAR3 receptors prefer saturated or ali-cyclic moieties of SCAs for ideal binding [51]. Histidine at 4-TM (His^140^) and 6-TM (His^242^) is important in deciding the binding efficacy of SCFAs in human FFAR3, as indicated by mutagenesis studies replacing these AAs with alanine [128,134]. SCFAs via FFAR3 regulate various biochemical, cellular, and physiological function such as metastasis, hormone synthesis, gut motility, adipogenesis, lipolysis, apoptosis and others [119,121,123,126]. Detailed in-silico analysis on the binding efficiency of SCFAs with FFAR3 in mice, rodent or any other species needs to be studied.

Although the interactions of SCFAs with FFAR2/3 are similar, they still show a degree of selectiveness in these interactions [11,50]. In addition, these complex interactions can be resolved by designing alternative compounds that show higher efficacy and selectiveness for binding [51]. However, more comprehensive studies are required to define the biological functions of FFAR2/3 independent of compensative effects. There is also critical need to develop specific compounds for activating FFAR2/3 with higher efficacy than SCFAs to exploit their therapeutic potential [50]. The following section describes a few examples of synthetic compounds that bind with FFAR2/3.

## 5. Interaction of Synthetic Ligands with FFAR2/3

### 5.1. FFAR2 Interaction with Synthetic Ligands

**Figure 5:**
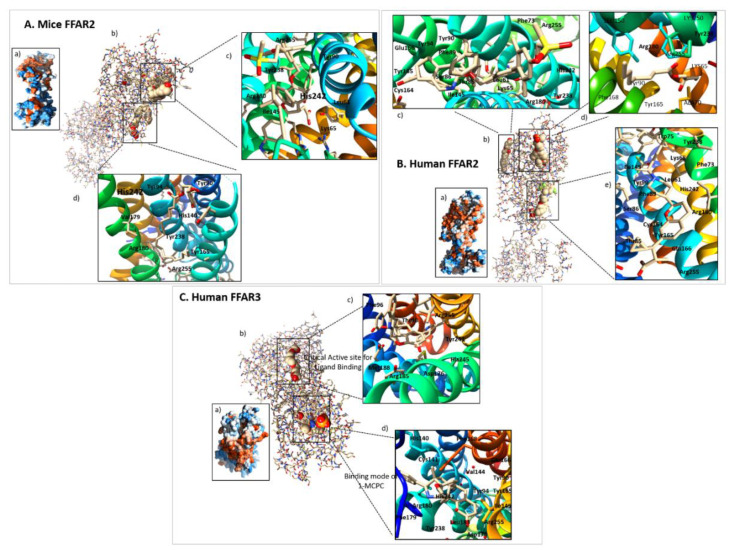
Mice (**A**) and human (**B**,**C**) FFAR2 (**A**,**B**) and FFAR3 (**C**) Protein-ligand interaction at orthosteric and allosteric sites. (**A**) Mice FFAR2 protein-ligand interaction (a) Hydrophobic model; (b) Ball and stick model with (c) Orthosteric binding site of C3 (Propionate), (d) Allosteric binding site of Cmp1; (**B**) Human FFAR2 protein-ligand interaction (a) Hydrophobic model, (b) Ball and stick model with (c) Orthosteric binding site of 4-CMTB, (d) Critical binding sites, (e) Allosteric binding site of 4-CMTB; and (**C**) Human FFAR3 protein-ligand Interaction (a) Hydrophobic model, (b) Ball and stick model, (c) Critical active sites for ligand binding, (d) Binding mode of 1-MCPC.

Synthetic ligands such as CATPB and GLPG0974 act as allosteric antagonists to the FFAR2 receptor by reducing Ca^2+^ and the phosphorylated extracellular signal-regulated kinase (ERK)1/2 pathway [137,138,139]. For the first time, CATPB interaction with human and mouse FFAR2 are shown in Figure 4. Synthetic ligands such as Compound 1 (Cmp1), ZINC03832747, compound 44, phenylacetamide 58, and Euroscreen compound series are orthosteric agonist of human FFAR2 [128,140]. Cmp1 is also an orthosteric agonist for mice FFAR2 [128,137]. Along with mutation at His^242^ site to alanine, mutation within the binding pockets at His^140^, Val^179^, Tyr^90^, Tyr^165^, and Tyr^238^ residual sites to alanine significantly reduced agonist property of Cmp1 for mice FFAR2 [141], as shown in Figure 5A.

*DEFINITIONS:*

***Orthosteric ligands***. The ligand which binds to a receptor at an endogenous active site.

***Allosteric ligands***. The ligand which binds to a receptor other than an endogenous site.

***Allosteric agonist ligands***. Allosteric ligand that activate the receptors in the absence of orthosteric ligands by binding other than on an active site.

***Ago-allosteric ligands***. The ligand binds allosterically to activate a receptor in the absence of an orthosteric ligand equal to an allosteric agonist and also activates the receptor in the presence of an orthosteric ligand as a positive allosteric modulator (potentiate agonist-mediated receptor response).

***Inverse agonist***. An inverse agonist is a ligand that binds to the same receptor as an agonist but induces a pharmacological inhibitory response.

***Inverse agonist***. The ligand binds to the receptor as an agonist but develops pharmacological function opposite to that of agonist.

The CFMB (previously known as phenylacetamide 1) [142], AMG-7703 [143], and tiglic acid [125] are allosteric agonists (that bind to other than orthosteric sites and activate receptor activity) of human FFAR2. CFMB forms H-bond at Ile^66^, Phe^89^, Leu^173^, Tyr^238^, and Val^259^ residues [142], while AMG-7703 forms H-bonds at Ile^66^, Phe^89^, Leu^173^, Val^259^, Tyr^90^, Ile^145^, Asn^239^, and His^242^ [143] with human FFAR2. Histidine residue at site (His^242^) in human FFAR2 serves as a key residual site to classify whether a ligand will show allosteric or orthosteric activity [143]. 2CTAP, BTI-A-404, and BTI-A-292 are inverse agonists (a ligand binds to the receptor as an agonist but inhibits its pharmacological response) of human FFAR2 and reduced Ca^2+^ level via Gα_q_ signaling [120,144]. However, detailed information on the structural and molecular interactions of 2CTAP, BTI-A-404, BTI- 292, and GLPG0974 with human FFAR2 are not available.

4-CMTB is an ago-allosteric modulator ligand for human FFAR2 as it increases the binding efficacy of SCFAs (like positive allosteric modulators) and also activates the human FFAR2 receptor of its own (like an allosteric agonist) [120,143,145,146]. The ago-allosteric modulator 4-CMTB binding interaction with human FFAR2 receptor is shown in Figure 5B. CFMB, phenylacetamide 2, and phenylacetamide 58 are allosteric agonists to mice FFAR2, but only demonstrated through biological phenomenons. However, in-silico studies remain unknown [51,140,142]. The chicken FFAR2 homology model has shown that four active residues are responsible for binding of vorapaxar ligand to FFAR2 receptor [91]. Three more AAs, Tyr^246^, Met^80^, and His^182^ provide supports to these ligand-binding grooves [91].

### 5.2. FFAR3 Interaction with Synthetic Ligands

Well-known human FFAR3 agonist 1-MCPC forms H-bonds at different binding residues to activate the FFAR3 receptor shown in Figure 5C [52,125]. Pertussis toxin (PTX) is a human FFAR3 inhibitor known to inhibit the FFAR3 receptors’ pharmacological and biological function via p38 and the c-Jun N-terminal kinase (JNK) pathway [124]. Similarly, based on biological phenomena, AR420626 and cyclopropanecarboxylic acid are selective allosteric agonists, and AR399519 and CF_3_-MQC are antagonists for mouse FFAR3, however detailed in-silico analysis has yet to be done [3,51,147]. To the best of our knowledge, so far no studies have directly addressed interactions of synthetic ligands with human or rodent FFAR3, therefore this opens opportunities to study the topic in detail using dry and wet lab technologies.

Interestingly, in many instances, FFAR2 and FFAR3 activities are interchangeable and/or compensatory, this is because of similar chemical and structural characteristics. For example, substation of FFAR2 amino acid residues such as Glu^166^, Leu^183^, and Cys^184^ with corresponding FFAR3 residues such as Leu, Met, and Ala using site-directed mutagenesis favored the binding of FFAR3 agonists 1-MCPC and 3-pentenoic acid, while these compounds were not able to bind wild type FFAR2 [51]. However, in humans as well as in mice, the important source of SCFAs (known orthosteric ligands of FFAR2/3) is the host gut microbiota, which drives the next step to discuss the role of gut microbiota in SCFAs production in regulating the pharmacological and physiological function of the host body through FFAR2/3 signaling.

## 6. Gut Microbiome Produces FFAR2/3 Ligands-SCFAs

**Figure 6:**
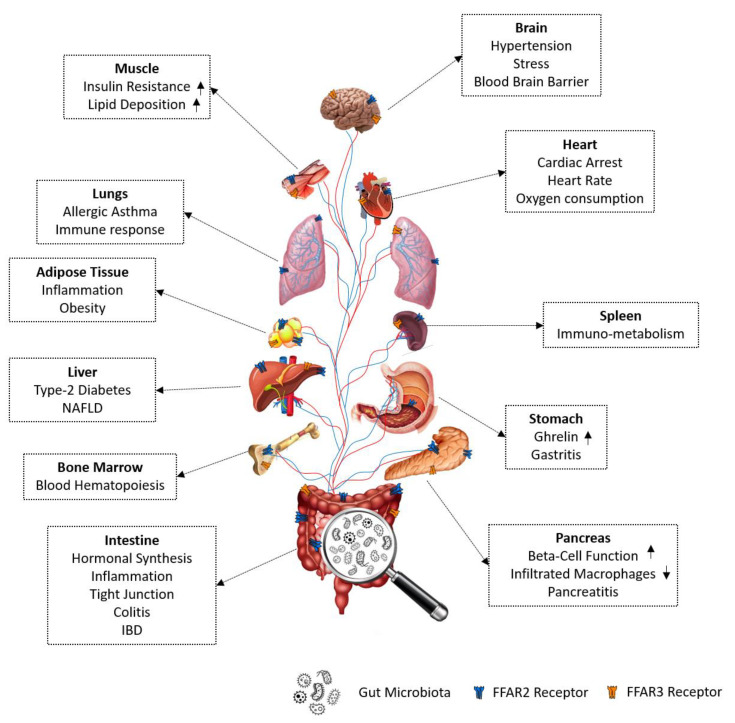
Biological function of FFAR2/3 as short chain fatty acid (SCFA)’s receptors at different body parts.

The gut microbiota is an important source of SCFAs that exhibit several health beneficial effects such as immune [35,44,56], metabolic [26,49,125,148,149], and neuronal [103,150] functions by activating FFAR2/3 signaling. The gut microbiota such as *Bacteroides (B.) thetaiotaomicron, Akkermansia (A.) muciniphila, Bifidobacterium *spp.,* Prevotella *spp.,* Ruminococcus *spp.,* Blautia hydrogenotrophica, Clostridium* spp., and *Streptococcus* spp. produce acetate from pyruvate via acetyl-CoA and/or the reductive acetyl-CoA pathway [150,151]. The propionate is produced by *B. thetaiotaomicron, Roseburia *spp.,* Firmicutes, Roseburia inulinivorans, Ruminococus *spp.,* Clostridiales (C.) Lacterium, Eubacterium (Eu.) *spp.,* Coprococcus *spp.,* Dialister succinatiphilus, Phascolarctobaterium succinatutens, A. muciniphila, Clostridium *spp.,* Coproccus catus, Clostridium *sp.,* Roseburia insulinivorans, Ruminococus* spp., and *Eu. halli* from succinate, acrylate and/or propanediol pathways [150,151]. Similarly, butyrate is produced by *C. tyrobutyricum, Roseburia intestinalis, Eu. rectale, Roseburia insulinivorans, Clostridiales bacterium, Anaerostripes hadrus, Coprococcus *spp.,* C. symbiosum, Faecalibacterium prasnitzii, Bacteroidetes* spp., and *Coprococcus* spp. by butyrate kinase and/or the butyryl-CoA:acetate CoA-transferase biosynthesis route [150,151]. The SCFAs produced by gut microbiota in the intestine not only act on local intestinal cells such as intestinal enteroendocrine cells [3], but also get absorbed from the gut and circulate through portal and systemic blood to act on cells including monocytes [22], white adipocytes [15], neurons [17,24], cardiac cells [23], hepatocytes [17,18], skeletal muscle [19,20], alveolar cells [6,21,22,152], pancreatic cells [16], bone marrow [10], and splenocytes [21,22]. One of the chief biological functions of SCFAs is activation ofFAR2/3 signaling, as these receptors are widely expressed in such cell types (Figure 6). Indeed, SCFAs ameliorate obesity [26,32,49,121], diabetes [27,29,153], and colitis [2,34,44] which involves activation of FFAR2/3, indicating that gut microbiota-derived SCFAs’ mediated activation of FFAR2/3 signaling plays a crucial role not only in maintaining normal physiological and cellular functions but also protecting from diseases.

## 7. Biological Functions Regulated by FFAR2/3 Signaling

The biological functions regulated by FFAR2/3 signaling are immunity [35,56,114,154], gut hormonal synthesis [11,24,144], gut integrity [78], and neuronal function [3,101] to maintain the body homeostasis [3,11,24,119,135] (Figure 6) and described below.

### 7.1. FFAR2/3 in Immune Regulation

#### 7.1.1. FFAR2 in Immune Regulation

In humans, FFAR2 exhibits an anti-inflammatory response against metabolic diseases [22,56,155]. FFAR2 agonist CFMB treatment reduced pro-inflammatory response in human monocytes by increasing phosphorylation of p38-mitogen-activated protein kinase (MAPK) signaling [22]. Moreover, FFAR2 knock-out (KO) mice show more severe inflammation in colitis, arthritis, and airway inflammatory (asthma) in mice, which indicates that FFAR2 signaling helps in reducing the proinflammatory response [35,40,156]. FFAR2 KO mice show enhanced neutrophil migration and proinflammatory cytokine secretion in the intestine [2,45,157]. Moreover, activation of FFAR2 by SCFAs ameliorates colitis in chronic dextran sodium sulphate (DSS)-induced colitis mice model [70] (Table 2).

FFAR2 signaling activation led to increase immunoglobulin (Ig)A (first line of defense against pathogens at the mucosal surfaces) production to protect intestinal epithelium against foreign pathogenic microbe invasion [115,158]. Sun et al. [153] showed that activation of FFAR2 signaling increases cathelicidin-related antimicrobial peptide (CRAMP) production from pancreatic endocrine cells as protection against type 1 diabetes (T1D) [153]. In addition, butyrate-mediated activation of FFAR2 signaling in mice chondrocyte exhibit anti-inflammatory activity by inhibiting the phosphorylation of NFκB (nuclear factor kappa-light-chain-enhancer of activated B cells), MAPK, AMPK-α (5’ adenosine monophosphate-activated protein kinase), and the PI3K (Phosphatidylinositol 3-kinase)/Akt (Protein Kinase B) pathway [159]. Moreover, FFAR2 activation by SCFAs reduce IECs graft-versus-host disease by activating nucleotide-binding oligomerization domain-like receptor protein 3 inflammasome (associated with IECs repairing by IL-18 secretion and maintaining integrity) [160]. FFAR2 also induces neutrophil chemotaxis through activation of P13Kγ, Rac2 (Rho family GTPase), p38-MAPK, and extracellular signal-regulated kinases (ERK) signal transduction pathway [161]. On the other hand, FFAR2 KO mice with chronic DSS-induced colitis phenotype shows a decrease in invasion of PMNs and cytokine keratinocyte chemoattractant synthesis as compared to wild-type (WT) mice [42,43,45]. Additionally, FFAR2 KO mice reveal decrease in adaptive inflammatory response as compare to WT littermate in gout pathology [39]. This debatable immune modulation by FFAR2 signaling needs further studies to understand its precise mechanism(s) and their importance in different disease pathologies.

#### 7.1.2. FFAR3 in Immune Regulation

FFAR3 signaling activated by acetate and propionate reduces the production of pro-inflammatory cytokine (Tumor Necrosis Factor alpha [TNF-α]) secretion [42], and enhances anti-inflammatory chemokines (C-X-C motif ligand 1 (CXCL-1) and CXCL-2) via enhancing the extra-cellular ERK1/2, p38-MAPK, PI3K, or mTOR (mammalian target of rapamycin) signaling [44,164,173]. In addition, FFAR3 expression increases on soluble fiber administration with a decrease in macrophages, eosinophils, neutrophils migration, and exhaled nitric oxide synthesis (eNOS) against asthma [40], so FFAR3 signaling enhances adaptive immune response. Moreover, in influenza infected mice, FFAR3 pathway increases anti-viral immunity activity on dietary fermentable fibers and SCFAs administration [114]. In addition, FFAR3 pathway stimulated by propionate reduces the lungs’ allergic inflammation and total amount of IgE (antibody associated with allergic reaction) concentration in the serum [6]. Moreover, FFAR3 KO mice show lower immune response against *Citrobacter rodentium* infection with delayed in expression of interferon gamma (INFγ) (critical cytokine for innate and adaptive immunity against infection) through rapidly accelerated fibrosarcoma which activates the MAPK/ERK pathway [44]. However, single-cell RNAseq of eosinophilic esophagitis patient T-cell exhibits higher expression of FFAR3 with increased Th2 cytokine (that exacerbate allergies) production [41,174]. These observations indicate that FFAR3 signaling is involved in differential immune response of allergic reactions.

In mice macrophages (Raw 264.7), activation of FFAR3 signaling by SCFAs reduces the proinflammatory cytokines and increases nitric oxide synthase (iNOS) secretion [48]. Moreover, in human umbilical vein endothelial cells (HUVEC), FFAR3 mediated signaling reduces the LPS or TNFα stimulated atherosclerosis by inhibiting the proinflammatory cytokines and vascular cell adhesion molecule-1 synthesis on propionate and butyrate treatment [62,175]. However, another study reported that butyrate treatment in femoral bone marrow derived macrophages develops an anti-microbial effect through histone deacetylases inhibitor (HDACi) pathway independent of FFAR3 [176]. There, epigenetic or FFAR2 immune response compensates FFAR3 immune signaling. However, in ruminant *Capra hircus* fed with a high concentrate diet (60%) increases LPS and SCFAs production that activate FFAR2/3 signaling to produce cytokines and chemokines which in turn lead to cecal inflammation [177]. These results indicated that the role of FFAR3 signaling in regulating inflammation is controversial, and it may be disease/context dependent, hence further studies are needed to comprehend the role of FFAR3 signaling in immune modulation in a disease-specific manner.

Overall, these observations indicate that both FFAR2/3 are closely associated with complex mechanism of immune response [42,70,161,178,179], and cell specific responses in different diseases remain to be elucidated.

### 7.2. FFAR2/3 in Gut Hormonal Synthesis

**Figure 7:**
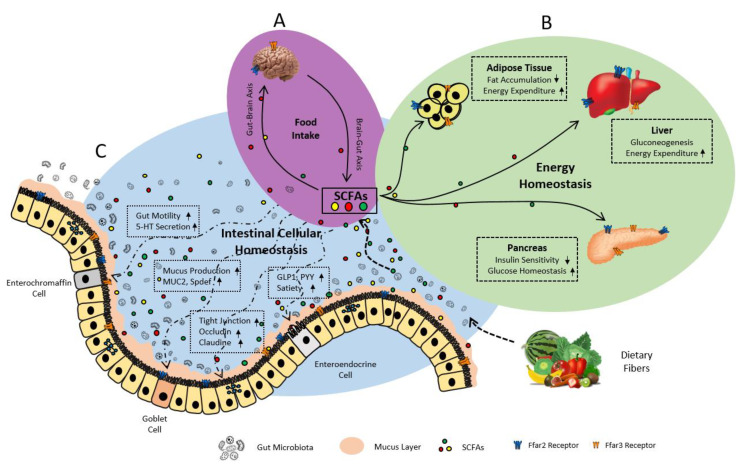
Role of diet-derived SCFAs activated FFAR2/3 signaling in regulation of energy balance through (**A**) Regulating the food intake by modulating gut-brain axis (**B**) Maintaining homeostasis by decreasing the fat accumulation and increasing the energy expenditure in adipose tissues, manipulating rate of gluconeogenesis in liver, and increasing insulin secretion and beta-cell function in the pancreas (**C**) Maintaining intestinal cellular homeostasis by increasing gut transit, mucus production, tight junction protein expression, and gut hormone synthesis and secretion.

FFAR2/3 signaling significantly contribute in gut hormone homeostasis through gut-hepatic [5,169] and gut-brain [3,150,180] axis regulate metabolic functions (Figure 7). Incretin hormones such as PYY and glucagon-like peptide 1 (GLP-1) secreted from L-cells (ileum and colon) have anorexigenic effect (reducing food intake) through enhancing expression of pro-opiomelanocortin (POMC) whereas suppressing agouti-related peptide (AgRP) and neuropeptide Y (NPY) in the hypothalamus of the brain. While ghrelin (secreted from X/A-like cells in stomach) acts as an orexigenic effect (increasing food intake) via increasing NPY/AgRP signaling [24]. Overall, FFAR2/3 play a vital role in maintaining homeostasis of neuropeptides (GLP-1, PYY, CCK, ghrelin) and neurotransmitters (catecholamine, serotonin, and GABA) synthesis, and nutrient absorption [24,121,169], and detailed evidences are described below.

#### 7.2.1. FFAR2 in Gut Hormone Synthesis and Secretion

FFAR2 activation increases GLP-1 and PYY synthesis in human, rodents and guinea pig L-cells [63,76,181,182]. Tolhurst et al. [11] for the first time reported that activated FFAR2 signaling increases GLP-1 hormonal synthesis from L-cells of mice with an increase in Ca^2+^ levels. In addition, FFAR2 KO mice show a decrease in GLP-1 and insulin secretion leads to impair glucose tolerance even under SCFA treatment [11]. However, inulin (a prebiotics that promotes SCFA production) feeding increases L-cell population in HFD-fed mice and protects against obesity/T2D, while such effects of inulin disappeared in FFAR2 KO mice [24], suggesting that FFAR2 is required for acetate action to prevent HFD-induced obesity/T2D. The activated FFAR2 controls blood glucose by increasing PYY and GLP-1 [11]. In addition, FFAR2 agonist (CFMB) treatment to mice intestinal organoid directs more PYY and GLP-1 secretions with reduced cyclic adenosine monophosphate (cAMP) levels [24,172]. The novel FFAR2 antagonists such as CATPB, BTI-A-404, and BTI-A-292 decreases the GLP-1 hormonal synthesis from NCI-H716 cells through downregulation of ERK, p38 MAPK, and NF-κB pathways [144]. These results profoundly indicate that FFAR2 signaling regulates GLP-1 and PYY secretion and may pave the way to consider FFAR2 as a therapeutic target against diabetes, because GLP-1 increase is beneficial in regulation of blood glucose levels. However, in a rat study, FFAR2 agonist CFMB had no effect on colonic GLP-1 hormonal synthesis [183], indicating that either CFMB is not agonist for rat FFAR2 and or it plays different role in rat intestines compared to that of humans and mice.

In addition, FFAR2 signaling also involved in the GI tract buffering, especially on the conjunction of stomach and duodenum where acid of stomach poured down in the duodenum. A study showing that FFAR2 agonist phenylacetamide 1 increases the duodenal HCO_3_^-^ secretion via activating the 5-HT_4_ receptor, and muscarinic M_1_ and M_3_ receptors [171], which therefore balances the acidity coming from stomach in the duodenum.

#### 7.2.2. FFAR3 in Gut Hormone Synthesis

Tolhurst et al. [11] also reported that GLP-1 hormone synthesis is regulated by FFAR3 signaling mediated through SCFA. GLP-1 and PYY significantly reduced in FFAR3 KO mice as compared to their WT littermates [4]. However, FFAR3 agonist (AR420626) increases GLP-1 release in mice colonic crypts [3]. In addition, co-administration of maltose and miglitol (α-glucosidase inhibitor) to mice increases plasma SCFA and GIP levels via FFAR3. This effect was not seen in FFAR3 KO mice [184]. Such effect of dietary fibers on GLP-1 levels was not seen in antibiotic treated, germ-free and FFAR3 KO mice [184]. Activation of FFAR3 signaling increases the GLP-1 [11], while AR420626 (FFAR3 agonist) and AR399519 (FFAR3 antagonist) treatment to rats and AR420626 (FFAR3 agonist) treatment to mice intestinal organoid shows no effect on the synthesis of PYY and GLP-1 hormonal synthesis [24,183]. The exact reason behind these discrepant results is not known. Moreover, FFAR3 agonist (AR420626) treatment reduces enteropathy (ulcer formation and gastrointestinal bleeding) symptoms induced by indomethacin in rats by increasing duodenal HCO_3_^-^ and GLP-2 hormonal synthesis whereas FFAR3 antagonist (CF_3_-MQC) counteract the AR420626 effect by reducing the enteropathy condition [147], indicating that the mucosal protective effect of AR420626 was mediated by FFA3 activation.

Even so, increased synthesis of PYY and GLP-1 hormone by Roux-en-Y gastric bypass (RYGB) surgery leads to overexpression of both FFAR2 and FFAR3 in the intestine [185]. This indicates that incretin hormonal synthesis is associated with FFAR2/3 receptor signaling in response to metabolic syndrome in either direction. Either through genetic mutational study on FFAR2/3 or their interactive action with targeted agonists and antagonists would help in exploring the exact mechanism of action of FFAR2/3 against various gut hormonal comorbidities such as obesity and T2D.

### 7.3. FFAR2/3 in Intestinal Epithelial Integrity and Inflammation

Emerging evidence indicates that FFAR2/3 signaling significantly contributes to nutrient absorption [63,76] and helps to maintain intestinal epithelial integrity [8,24] (Figure 7), as described below.

#### 7.3.1. FFAR2 in Intestinal Epithelial Integrity and Inflammation

FFAR2 signaling contributes to (i) maintaining intestinal integrity which in turn reduces leaky gut, and (ii) regulating the colonic motility through intestinal 5-hydroxytryptamine (5-HT) release [66,76] and gut dysbiosis [66,76]. FFAR2 activation increases the expression of tight junction proteins (tight junction protein 1 [Tjp1], Occludin [Ocln], Claudine [Cldn]1), and mucus-secreting markers such as mucin (Muc)1 and Muc2 to maintain intestinal integrity [186], thereby reducing pro-inflammatory markers (interleukin [IL]-1β and TNF-α). In contrast, a significant decrease in mucin production (Muc2, Muc3, Muc4, and -Muc5b) was observed in the intestine of FFAR2 KO mice further indicating that FFAR2 KO mice have compromised gut barrier functions that were associated with reduced antimicrobial peptide synthesis (Reg3α, Reg3β, and Reg3γ), suggesting higher risk of microbial translocation [156]. Even in chickens, the modulated intestinal microflora by galactooligosaccharides increases the intestinal innate immune response and barrier function along with FFAR2 gene expression, suggesting that FFAR2 receptors are involved in maintaining intestinal immune homeostasis [187]. However, antibiotic treatment reduces FFAR2 expression and increases colonic epithelial permeability and inflammatory cytokines (TNF-α and IL-10) [78] in mice, further suggesting the role of FFAR2 in maintaining intestinal homeostasis. In addition, the FFAR2 KO mice model with dextran sodium sulphate (DSS)-induced colitis exhibits a decrease in colon length, an increased morbidity, an increased daily activity index (DAI), the inflammatory mediator myeloperoxidase, and a decrease in innate immunity markers such as toll-like receptors (TLR2 and TLR4) compared to their control-FFAR2WT littermate [35,71,154,157,183,188]. While FFAR2 agonist reduces body weight gain, DAI, fecal Lipocalin-2 level (biomarker of intestinal inflammation), and pro-inflammatory cytokines (IL-6) and keratinocytes chemoattractant cytokine secretion from colonic mucosa of DSS-induced colitis mice, suggesting that FFAR2 agonism protects against colitis [71].

Also, FFAR2 KO-NOD mice have a higher rate of T1D development as compared to FFAR2 WT-NOD mice [178]. However, acetylated high-amylose maize starch administration to FFAR2WT-NOD mice shows protection against diabetes but such effect was no seen in FFAR2 KO-NOD mice [178]. Furthermore, butylated high-amylose maize starch administration to FFAR2 KO-NOD mice show protection against diabetes due to an increase in the population of CD4+Foxp3+ Treg cells in the colon [178]. At the molecular level, via epigenetic-histone modification butyrate converts the naive Fox3^-^ T-cells to Fox3^+^ Treg cells through overexpression of FoxP3 protein, IL-10 and Helios transcription factor to provide protection against T1D (or autoimmune activity) by increasing the number of autoreactive T cells and Treg cells [178]. In human intestinal PBMC, FFAR2 agonist butyrate reduces gut permeability and protection against LPS-induced pro-inflammatory (IL-1β and TNFα) production [8,189]. SCFAs reduce colonic inflammation by decreasing the secretion of proinflammatory cytokines (IL-6 and IL-12), and chemokines from the intestinal epithelial cells and/or through increasing IgA and IgG (B cells) production and interacting with DCs in TNBS (2, 4, 6-trinitrobenzene sulfonic-acid- an intestinal inflammatory agent) and *C. rodentium* infection induced intestinal inflammation in FFAR2 KO mice [44,190]. However, inulin (a dietary fiber) feeding, which increases SCFAs (ligands of FFAR2), also increased the expression of tight junction proteins independent of FFAR2/3 [24]. In addition, activated FFAR2 signaling by natural indigenous fruit black raspberries increases the host immune response in gut of human and colorectal cancer mice (Apc^Min/+^) model [191,192]. However, contradictory findings reported by Hatanaka et al. [193] showed that the FFAR2 signaling promotes occurrence of GIT tumorigenesis. This controversial result of FFAR2 in intestinal integrity might be due to (i) epigenetic changes induced by SCFAs and/or (ii) a compensatory response by FFAR3 signaling. Further, precise mechanistic studies to develop full understanding about the role of FFAR2 signaling in intestinal integrity are warranted.

#### 7.3.2. FFAR3 in Intestinal Epithelial Integrity and Inflammation

FFAR3 maintains intestinal integrity by activating the cytokines and chemokines through the MEK-ERK pathway [44]. In FFAR3^-/-^ mice, the inflammatory response was significantly reduced as compare to their WT [44]. Grape seed proanthocyanidins reduces the diarrhea occurrence by improving intestinal integrity and by shifting towards SCFAs-producing microbes (*Lactobacillaceae and clostridium*) in young swine models [194]. SCFAs also decrease intestinal permeability by increasing Ocln and FFAR3 mRNA expression in swine intestine [194]. The SCFAs treatment shows potential inhibitor action against LPS-induced pro-inflammatory (IL-1β, IL-6 and TNFα) production by activating FFAR3, tested in FFAR3 KO mice signaling [8,44,189]. However, FFAR3 KO mice on treatment with TNBS shows reduced immune response along with suppression of neutrophil infiltration [44], so apart from FFAR3 signaling, intestinal inflammatory action is regulated by some other mechanism.

### 7.4. FFAR2/3 in Neurophysiology

After the deorphanization, many research groups have reported that neither FFAR2 nor FFAR3 are expressed in CNS [9,101]. However, recently it has been reported that low expression of FFAR2 is detected in the CNS which is limited to glia and neurons of the caudate, but FFAR2 can also be detected in cortical neurons and pituitary gland [33]. FFAR3 is expressed in PNSs, particularly sympathetic neurons of the superior cervical ganglion as a vasoconstriction phenotypic effect [23,101,102].

#### 7.4.1. FFAR2 in Neurophysiology

FFAR2 regulates the blood brain barrier (BBB) permeability [150]. Butyrate-mediated activation of FFAR2 signaling and colonization of single bacterial strain *Clostridium tyrobutyricum* (responsible for production of butyrate) and *Bacteroides thetaiotaomicron* (mainly produce acetate and propionate) in germ-free mice decreases BBB permeability through boosting Ocln mRNA expression in the frontal cortex and hippocampus [150]. Even FFAR2 KO mice show severe microglia abnormality with increased dendritic lengths, number of segments, branching points, terminal points, and cell volumes as compared to control mice suggesting that FFAR2 regulates microglial maturation and function [9]. However, multiple sclerosis (autoimmune neuro-inflammatory disease associated with CNS) patients and experimental autoimmune encephalitis (EAE) mice models induced by immunization of myelin oligodendrocyte glycoprotein show lower SCFA concentration and a high expression of proinflammatory marker along with FFAR2 and 3 expression [195]. This is further supported by clinical and histological score that the FFAR2/3 KO mice are more resistant to experimental autoimmune encephalitis (EAE) pathogenesis as compared to WT mice [195]. However, administration of SCFAs to EAE mice shows an anti-inflammatory effect by increasing the IL-10+ T-cells and IL-10 expression in CNS tissues to suppress the inflammation. Thus, despite SCFAs’ beneficial effects on the CNS function, the mechanisms of SCFAs and FFFAR2/3 signaling to protect autoimmune CNS inflammation are not known [195]. As SCFAs also function through inhibition of histone deacetylase and modulating cellular energy flux such as mitochondrial functions, this may be responsible for such effects. However, these pathways are not yet established in EAE pathogenesis.

#### 7.4.2. FFAR3 in Neurophysiology

FFAR3 controls sympathetic neurons which in turn regulate whole body metabolic homeostasis [23]. FFAR3 is expressed in portal neurons and regulates propionate-induced gluconeogenesis via gut-brain axis [149]. In FFAR3 KO mice, catecholamine-producing enzyme tyrosine hydroxylase (TH) level is significantly lower which affects the neuronal growth [23]. The heart rate is reduced in FFAR3 KO mice which is associated with decreased norepinephrine release from sympathetic neurons, indicating that FFAR3 signaling regulates sympathetic neuronal functioning [23]. FFAR3 signaling activates G_βγ_-phospholipase C (PLC)-β3-ERK1/2-synapsin 2-β at serine 426 pathway to enhance norepinephrine release from sympathetic nerve endings [103]. FFAR3-dependent synthesis of norepinephrine releases from synaptic vesicles which helps to modulate energy expenditure of the host body [103]. Further, the treatment of mice with FFAR3 agonist propionate, elevates the heart rate and oxygen consumption by increasing β-adrenergic receptor in ganglions [23]. In addition, FFAR3-signaling inhibit N-type calcium channels in neurons [102,196].

Won et al. [104] suggests FFAR3 signaling activates G_βγ_ signaling pathway and inhibits N-type Ca^2+^ channels, which in turn reduces neuronal catecholamine release in rat sympathetic nervous systems. Moreover, in proximal colonic mucosa of rats, FFAR3 is associated with cholinergic-mediated secretory response in enteric nervous system [197]. Thus, FFAR3 is a potential target for treating neurogenic diarrheal disorder by reducing the nicotinic acetylcholine receptor (nAChR) activity [198]. Moreover, on treatment with FFAR3, synthetic agonist AR420626 suppresses nAChR or serotonin mediated motility changes with a consistent effect on the FFAR3-stimulated anti-secretory effect [198]. FFAR3 expressing neurons in sub-mucosal and myenteric ganglionic plexus of small intestine regulates gut hormonal synthesis [3]. Mostly in the distal part of small intestine (ileum), the FFAR3-expressing neurons reported to be expressed in substance P and somatostatin enteroendocrine cells derived from the CCK-secretin-GIP-GLP1-PYY-neurotensin lineage [3,180]. These evidence shows FFAR3 signaling similar to FFAR2 is a promising therapeutic target for treating gut related disorders such as obesity, T2D, colitis and diarrhea, by honing gut-hormonal synthesis and balancing the microbiome-gut-brain axis (Table 3).

### 7.5. FFAR2/3 in Adipogenesis and Lipolysis

Several cellular and molecular pathways involved in adipogenesis, lipolysis, glucose homeostasis, insulin sensitivity, and energy metabolism are regulated by FFAR2/3 signaling (Figure 7) [3,11,28,30,75,121,148]. FFAR2/3 signaling prominently modulates leptin secretion from adipose tissue to impact adipogenesis and dysglycemic conditions [119,148].

#### 7.5.1. FFAR2 in Adipogenesis and Lipolysis

FFAR2 is responsible for energy accumulation in adipose tissues, adipogenesis, and metabolic syndrome disease pathogenesis [113]. In-vitro (differentiated 3T3-L1 cells) and in-vivo (C57BL/6J mice) study on adipocyte has shown that FFAR2 increases adipogenesis [75]. In mice, acetate and propionate administration boosts FFAR2 expression in adipose tissues with reduce plasma FFA levels and decrease lipolysis [31,75,140]. Moreover, when FFAR2 KO mice fed with high fat diet (HFD) show higher energy expenditure, plasma FFA level and higher food intake leads to obesity as compared to WT mice [30,33,117]. However, activated FFAR2 signaling by SCFA administration to diet-induced obese (DIO) mice demonstrates reduced body weight by promoting beige adipogenesis and mitochondrial biogenesis with reduction of *Firmicutes: Bacteroidetes* ratio along with lower plasma FFA level [19,199]. Moreover, SCFA treatment to adipose-specific FFAR2 KO transgenic (aP2-Gpr43TG) mice induces pro-inflammatory cytokine (TNF-α) in anti-inflammatory M2-type macrophages within the adipose tissue milieu [200]. Apart from SCFAs, ketogenic metabolites aceto-acetate activates FFAR2 which activates ERK1/2 signaling in ketogenic condition (fasting or diabetic) to regulate energy homeostasis and maintains lipid metabolism [201]. During lactation, bovine adipocytes exhibit higher FFAR2 expression, which indicates genetic switch-on of FFAR2 enhanced adipogenesis to compensate for high energy requirement of the animal during lactation [84].

**Table 3:** Physiological function of FFAR3 in humans and mice/rodents.

S.No.	Tissue/Organ	Research Findings	Ref.
**Human**			
1	Intestinal L- cells	-Secrete GLP-1 and PYY in response to glucose	[3,11,76]
2	Intestinal I- cells	-Secrete Cholecystokinin (CCK) in response to glucose.	[3]
3	Intestinal K- cells	-Secrete glucose-dependent insulinotropic peptide (GIP) in response to glucose.	[3]
4	Colon	-No effect of propionate response on Intestinal Gluconeogenesis (IGN) genes (G6PC, PCK1, MUT) expression with either FFAR2 agonist such as tiglic acid (TA) or FFAR3 agonist i.e,1-methylcyclopropanecarboxylic acid (MA). -IGN gene expression increases by butyrate mediated through cAMP pathway but not via G_i_- nor G_q_ pathway. -Neither Gi- nor Gq-sensitive inhibitors (PTX and U73122) able to reduce the IGN gene expression induced by butyrate.	[149]
5	Monocyte	-Human monocyte FFAR3 reduces cytokine expression in response to acetate. -The receptor modulates p38-MAPK signaling in response to acetate and FFAR3 agonist (AR420626).	[22]
6	Adipocytes	-FFAR3 expressed in the human multipotent adipose tissue-derived stem cells (hMADS). -Acetate is responsible for the antilipolytic response luminal and systemic level. -Rosiglitazone increases the expression of FFAR3. -FFAR3 stimulation develop anti-inflammatory action targeting TNFα and IL-1β. -Treating with G_i_-sensitive PTX inhibitors prevents antilipolytic response develop by acetate. -Colonic or systemic acetate modulation helps in improving the insulin resistance in human adipocytes via FFAR3 mediated attenuation of hormone-sensitive lipase (HSL) phosphorylation.	[15,202]
7	Enteric Neurons	-FFAR3 agonist, AR420626 response at colon mucusa showed monophasic reductions in short-circuit currents (Isc) and sensitive to neurotoxin tetrodotoxin (TTX). -At submucosal and myenteric neuronal plexus, the FFAR3 is colocalized with Vasoactive intestinal polypeptide (VIP). -FFAR3 antagonist AR399519 inhibits FFAR3 agonism activity in entire colonic region.	[3,165]
**Mouse/Rodent**			
1	Pancreatic α- and β-cells	-FFAR3 is transcribed from the promoter of the GPR40. -The expression is mediated via an internal ribosomal entry site (IRES) located in the intergenic region of a bicistronic mRNA. -Helps in proper understanding in the identification of therapeutic target.	[16,28,73,203]
2	Primary Pancretic Islet	-FFAR3 expression in murine pancreatic islet -Leads to reduction of insulin secretion by coupling to Gi-type G Proteins in type-2 diabetic condition. -Locally to islet as well as in systemic circulation acetate concentration increases. -So, in type-2 diabetic condition FFAR3 antagonist may increase insulin secretion	[16,28,168]
3	Primary Pancreatic Islet	-Infusion of Acetate, propionate and butyrate has no profound effect on insulin and glucagon secretion regardless of glucose level. -Whereas, FFAR3 agonist Compound 4 (N-(2,5-dichlorophenyl)-4-(furan2-yl)-2-methyl-5-oxo-1,4,5,6,7,8-hexahydroquinoline-3-carboxamide) has significant effect in increasing the somatostatin and insulin secretion but showed no effect on glucagon synthesis.	[168]
4	Sympathetic Nervous System (SNS)	-Expressed in the rodent SNS especially at Superior cervical ganglia (SCG) and Celiac-mesenteric Ganglia (CSMG). -Induced variable I_Ca_^2+^ modulation activity by sodium propionate in the FFAR3^+/+^ mice. -Moreover, along with acetate and propionate, ketogenic metabolites β-hydroxybutyrate (BHB) produced voltage dependent reduction of N-type Ca^2+^ channel in SNS. -FFAR3-expressing neurons from reporter mice expressed decrease in Ca_v_2.2-FFAR3 inhibitory coupling variability. -FFAR3 is expressed primarily in neurons with a vasoconstrictor phenotype.	[101,102,104]
5	Superior cervical ganglia (SCG)	-Propionate enhances the norepinephrine (NE) release from primary-cultured mice SCG. -Pretreatment with G_i/o_ pathway sensitive-PTX; Gβγ inhibitor-Gallin; PLC inhibitor U73122 and MEK inhibitor U0126 significantly reduces NE secretion indicating the involvement of G_i/o_, Gβγ, PLCβ3 pathway in hormonal secretory function. -Treatment with Gα_i/o_ inhibitor NF023 shows no inhibition of NE release, so FFAR3 response is independent of Gα_i/o_ pathway. -Further, treatment with siRNA against either PLCβ3 or ERK1/2 decreases the expression NE protein by more than 80%. -So, SCFA receptor FFAR3 is coupled with G_i/o_ protein, to release NE via Gβγ-PLCβ3- ERK1/2-synapsin 2 pathway.	[103]
6	Intestine	-IGN induction is mediated by propionate through gut-brain axis. -Dietary propionate leads to c-Fos (neuronal activation marker) activation in the hypothalamic region which receives neuronal signal from both parabrachial nucleus (PBN) and dorsal vagal complex (DVC), mostly paraventricular nucleus (PVN), the lateral hypothalamus (LH) and the arcuate nucleus (ARC) of hypothalamus.	[149]
7	Intestinal Enteroendocrine Cells	-Acetate, propionate and butyrate administration in mice protect against diet-induced obesity and insulin resistance. -Propionate and butyrate but not acetate induce gut hormones and reduces food intake. -Butyrate had minor effect in stimulation of GLP-1 through FFAR3. -FFAR3 KO mice shows normal body weight and glucose homeostasis, indicating some additional mediators are involves in these mechanism. -FFAR3 KO mice shows impair GLP-1 synthesis with altered in mRNA expression of Glucagon, PYY and active GLP-1 peptide.	[11,121,148,204]
8	Monocytes	-Mice monocyte shows increase in IL-1α, IL-1β and GM-CSF cytokine expression in response to acetate. -Even in FFAR2/3 KO mouse monocyte displays elevate cytokine response on treatment with SCFAs. -So, SCFA does not act through FFAR2 to modulate mice monocyte inflammatory responses.	[22]
9	Neutrophil	-FFAR3 pathway is associated with airway neutrophil response subjected to influenza infection verified in FFAR3 KO mice.	[114]
10	Bone marrow	-FFAR3 KO mice produce less monocytes and interstitial macrophages from the bone marrow in response to butyrate	[114]
11	Ileum and Colon	-Moreover, dietary (Flaxseed) fibers restructured the gut microbiota with proliferation of the genera *Bifidobacterium* and *Akkermansia* reduces fat mass and show improve tolerance to intraperitoneal and oral glucose via FFAR3. -Microbiota is associate with increase SCFA production acting through FFAR3 signaling. -Through selective FFAR3-agonist, AR420626 showed greatest efficacy of FFAR3 at distal regions of intestine to protect mice from diet induced obesity by preventing a reduction in energy expenditure induced by an HFD.	[148,165,198]
12	Colonic Mucosa	-FFAR2 express in the colonic mucosa -Withdrawal of ceftriaxone antibiotic leads to reduction in SCFA concentration and increase number of conditionally pathogenic *Enterobacteria*, *E. coli*, *Clostridium*, *Staphylococcus spp*. and hemolytic bacteria in colonic gut. -FFAR2 immune regulation mechanism get hampered with increase in cytokine concentration in colonic mucosa. -Increased histopathology condition of colitis with goblet cell dysfunction, colonic dilatation and wall thickening, ultimate leads to IBD.	[78]
13	Duodenum L- cells	-FFAR3 is colocalized with GLP1 and expressed in L cells. -SCFAs (mostly acetate) activate FFAR2 and FFAR3 followed by 5-HT and GLP-2 release.	[171]
14	Enteric Neurons	-FFAR3 agonism (by AR420626) at descending colon mucusa was inhibited by neurogenic sensitive tetrodotoxin (TTX). -FFAR3 agonist activity is sensitive to acetylcholinergic (ACh) neurotransmission in rat colon mucosa. -ACh muscarinic antagonist atropine, nicotinic sensitive hexamethonium, FFAR3 antagonist AR399519, GLP1 antagonist Ex(3-39) or calcitonin gene related peptide (CGRP) blocker BIBN4096 abolished FFAR3 agonism activity in mouse colon region.	[3,165,197]
15	Stomach	-By qrtPCR and immunohistochemistry showed the expression of FFAR3 in villi and microvilli of gastric brush cells of mice stomach.	[3,7,172]
16	Enteric mucosal and submucosal cholinergic neurons of rat	-Suppresses carbachol (CCh)- or luminal propionate-induced Cl^-^ secretion influenced by TTX, hexamethonium and MQC through nicotinic ACh receptor activation. -SCFA-FFAR3 pathway responsible for anti-secretory function inhibited through cholinergic neural reflexes. -Pretreatment with serosal PTX along with MQC application restored the CCh response indicating the FFAR3 anti-secretory effect is mediated through G_i/o_ pathway in rat proximal colon.	[197]
17	Adipocytes	-A mixture of SCFA reduces plasma FFA in DIO mice along with beige adipogenesis marker. -Increase in adipose tissues with reduction in colon size. -Reduction in *Firmicutes: Bacteroidetes* ratio. -Reduces body weight by increasing mitochondrial biogenesis and reducing chronic inflammation.	[19,199]
18	Lungs	-Expressed in the mice lungs. -Propionate minimize allergy airway inflammation in mice lungs mediated through FFAR3.	[6]
19	Duodenal I-cells	-The receptor senses the circulating SCFA in plasma to modulate I-cell functions. -But unlike the LCFA, SCFAs are not involved in the cholecystokinin synthesis from duodenal I-cells.	[205]

FFAR3: Free fatty acid receptor 3; GLP-1: Glucagon-like peptide 1; PYY: Peptide YY; CCK: Cholecystokinin; GIP: Glucose-dependent insulinotropic peptide; IGN: Intestinal Gluconeogenesis; TA: Tiglic acid; MA: Methylcyclopropanecarboxylic acid; hMADS: human multipotent adipose tissue-derived stem cells; PTX: Pertussis toxin; HSL: Hormone-sensitive lipase; Isc: Short-circuit currents; TTX: Tetrodotoxin; VIP: Vasoactive intestinal polypeptide; IRES: Internal ribosomal entry site; Compound 4: (N-(2,5-dichlorophenyl)-4-(furan2-yl)-2-methyl-5-oxo-1,4,5,6,7,8-hexahydroquinoline-3-carboxamide); SNS: Sympathetic nervous system; SCG: Superior cervical ganglia; CSMG: Celiac-mesenteric Ganglia; BHB: β-hydroxybutyrate; NE: Norepinephrine; PLC: Phospholipase C; MEK: Methyl ethyl ketone; siRNA: Small interfering ribonucleic acid; PBN: Parabrachial nucleus; DVC: Dorsal vagal complex; PVN: Paraventricular nucleus; LH: Lateral hypothalamus; ARC: Arcuate nucleus; KO: Knock-out; TTX: Tetrodotoxin; ACh: Acetylcholinergic; CGRP: Calcitonin gene related peptide; CCh: Carbachol; MQC: N-[2-methylphenyl]-[4-furan-3-yl]-2-methyl-5-oxo-1,4,5,6,7,8-hexahydroquinoline-3-carboxamide; FFA: Free fatty acid; LCFA: Long chain fatty acid.

Additionally, in the differentiated 3T3-L1 cells, FFAR2 activation by propionate enhances adipogenesis via peroxisome proliferator-activated receptor gamma 2 (PPAR-γ2) pathway and the presence of FFAR2 siRNA (small interfering ribonucleic acid) which inhibits the adipogenesis process [75]. In addition, in 3T3-L1 cells, FFAR2 allosteric agonist (phenylacetamide 1 and 2) suppresses the adipocyte lipogenesis indicating activated FFAR2 receptors that reduces lipogenesis [140,142]. Moreover, in immortalized brown adipocyte cell lines (IM-BAT), Rosiglitazone (anti-diabetic adipogenic drug) increases the FFAR2 expression via PPARγ-dependent manner to regulate adipogenesis [32]. In contrast, FFAR2 KO mice fed with HFD show lower body fat mass, improved glucose control, lower plasma lipids, increased body temperature with BAT density, and lower WAT inflammation—indicating that FFAR2 deletion protects HFD-induced obesity/T2D [33,206]. Other studies show that acetate and propionate has no effect on adipogenesis in 3T3-L1 cells or mouse models and also no effect on either FFAR2 or FFAR3 expression [113,119]. A human study also reveals that FFAR2 expression in adipose tissues has no correlation with adipogenesis [207]. These observations indicate that the role of FFAR2/3 in adipose biology remain controversial and need further investigations.

#### 7.5.2. FFAR3 in Adipogenesis and Lipolysis

A human multipotent adipose tissue-derived stem (hMADS) model reveals that activated FFAR3 by acetate significantly reduces lipolysis through decreasing hormone-sensitive lipase phosphorylation [15]. In mice, FFAR3 stimulated by gut microbiota derived SCFA increases in leptin production, hepatic lipogenesis, and adipocyte adipogenesis [4]. Under HFD administration, FFAR3 KO male shows high body fat mass, plasma leptin level, and blood glucose level as compare to female littermates [204]. In pigs, stimulated FFAR3 by butyrate administration enhances lipid accumulation and adipogenesis by upregulating glucose uptake and de novo lipogenesis through activation of Akt and AMPK pathways [87]. Moreover, FFAR3 signaling reduces blood pressure of the mice by increasing renin (angiotensin secreted from kidney in controlling blood pressure, and maintaining body fluid and electrolytes level) production [20]. Furthermore, FFAR3 triggered by SCFAs regulates intestinal gluconeogenesis via cAMP-activated pathway [149] and satiety signaling through gut-brain axis [17,24], thereby controlling whole body energy metabolism. Moreover, butyrate effects to regulate lipolysis depends on FFAR3, as PTX (known FFAR3 antagonist) treated 3T3-L1 (adipocytes) and Raw 264.7 (macrophages) show no effects on lipolysis, while butyrate alone increases lipolysis in these cells [48]. The leptin synthesis and FFAR2 expression found low in adipose tissues of FFAR3 KO mice [74], however, reason for these changes are not known. Overall, these findings indicate that FFAR3 plays a significant, but controversial role in regulating energy metabolism, however, precise mechanism(s) remain elusive and need further investigations.

### 7.6. FFAR2/3 in Regulating Pancreatic Beta-Cells Proliferation and Functions

Pancreatic beta-cells are crucial to regulate blood glucose homeostasis by producing insulin. Therefore, maintaining and preserving beta-cell mass and functions remain critical. Beta-cell proliferation and differentiation is important for maintaining beta-cell population, while beta-cell functions are important for efficiently releasing insulin in response to glucose. In T2D, beta-cell proliferation, differentiation, and functions are deteriorated, which ultimately causes a decrease in insulin secretion and hyperglycemia in long-term diabetics. The coupling effect of FFAR2/3 receptors plays a fundamental role in the regulation of glucose-stimulated insulin secretion (GSIS) [28,39,73,203] and directly or indirectly responsible for β-cell functions in regulating pathology T2D [19,33,208].

#### 7.6.1. FFAR2 in Regulating Pancreatic Beta-Cell Proliferation and Functions

Starting from an early embryonic stage, maternal gut microbiota-SCFA-FFAR2 signaling plays a crucial role in regulating metabolic syndrome, as FFAR2 KO mice embryos have lower insulin and higher glucose level, and are more susceptible to obesity and diabetes in adulthood [209]. Additionally, FFAR2 KO mice on normal chow (NC) shows reduced β-cell mass and develop obesity and T2D characterized with increased glucose intolerance and FFA levels [30,32]. In addition, activation of mouse pancreatic β-cells- MIN6 by FFAR2 agonist (phenylacetamide 58) promotes proliferation and differentiation of β-cells and enhances insulin secretion [32,140]. In contrast, deletion of FFAR2 in Min6 and EndoC-βH1 cells (human pancreatic cell line) using siRNA increases the insulin synthesis [28]. Thus, the role of FFAR2 in regulating beta-cell proliferation, differentiation and their functions remains elusive and further comprehensive studies are needed to elaborate our understandings in this context.

#### 7.6.2. FFAR3 in Regulating Pancreatic Beta-Cell Proliferation and Functions

Gut microbiota changes in obese humans are associated with increased FFAR3/ Gi signaling to inhibit insulin synthesis [54]. These changes are further associated with epigenetic changes in FFAR3 receptor promotors (CpGs) and propensity of obesity and T2D, while lower methylation of FFAR3 promoters is associated with a higher body mass index [27]. However, FFAR3 activation by butyrate increases human β-cell mitochondrial respiration, which may be important to ameliorate beta-cell dysfunctions in T2D [127]. Additionally, in rodents, propionate stimulated FFAR3 signaling decreases the glucose oxidation and ATP/ADP ratio via the Gα_i/o_ pathway [49,210]. Opposite findings have been reported that either globally or pancreatic β-cell specific FFAR3 KO mice show greater insulin secretion and improvement of glucose tolerance [28]. Similar type of results reported in Min6 and EndoC-βH1 cell lines where FFAR3 antagonist (PTX) treatment increases insulin secretion [28].

Overall, these findings indicate that FFAR2/3 signaling is critical to regulate pancreatic beta-cells either by changing their proliferation, differentiation, insulin synthesis, and regulating their functions in terms of GSIS, which in turn maintains better glucose homeostasis, however, the their precise role in regulating proliferation and differentiation are poorly understood.

## 8. Conclusions and Future Directions

Dysbiotic gut microbiota with reduced SCFAs are related with suppression of FFAR2/3 signaling—that are known to regulate an array of biological pathways participation energy metabolism, adipogenesis, appetite control, intestinal cellular homeostasis, gut motility, glucose metabolism, and inflammatory response. Alterations in these biological pathways are hallmarks of several human diseases such diabetes, obesity, IBS/IBD, Crohn’s disease, atherosclerosis, gout, asthma, cardiovascular diseases, arthritis, hypertension, and colitis, therefore, targeting FFAR2/3 signaling can provide promising therapeutic strategies for these human diseases. The immune cell during metabolic diseases such as obesity and T2D causes chronic inflammatory response which provides an insight crucial mechanism for further disease progression. However, the role of FFAR2/3 signaling in these diseases remain controversial and needs to be further studied for better understanding of their role to devise the therapeutic importance of FFAR2/3 agonist/antagonists. For example, the majority of studies show that activation of FFAR2/3 signaling ameliorates obesity/T2D pathology, however, some studies show the opposite. For example, HFD feeding to FFAR2 KO mice shows improved oral glucose tolerance test (OGTT) and insulin sensitivity along with lower fat mass and increased lean mass compared to wild type (WT) mice [28,30,33], indicating that the deletion of FFAR2 protects HFD-induced obesity/T2D. Similarly, the mRNA and protein expression of FFAR2 has no correlation with insulin secretion in T2D patients [54,204]. Therefore, further studies are critically needed to develop better understanding about the role of FFAR2/3 in regulating metabolic functions, and pathology of obesity/T2D.

The pharmacological modification of these SCFAs receptors by endogenous or synthetic ligands provides an opportunity to counteract these gastrointestinal disorders in humans. However, overlapping expression of FFAR2 and FFAR3 in the same tissues/cells, and their similar affinity to specific endogenous ligands develop puzzled outcomes to understand the role of FFAR2/3 in particular biological functioning. Thus, the future studies must aim to develop highly specific and efficacious small molecules to modulate pharmacological actions of FFAR2/3 signaling, and can display a promising strategy to prevent, manage and/or treat human diseases including diabetes, obesity, Crohn’s disease, atherosclerosis, gout, asthma, cardiovascular diseases, arthritis, hypertension, and colitis.

## Figures and Tables

**Table 1:** Expression of FFAR2 and FFAR3 in different species’ cell lines.

S. No.	Cell Line/Type	Species	Tissues/Cells	Expression		Reference
				FFAR2	FFAR3	
1	3T3-L1	Mice	Adipose Tissue	Yes	Yes	Author *, [30,31,53,75,119]
2	3T3-L442A	Mice	Adipose Tissue	Yes	Yes	[53]
3	αTC1	Mice	Pancreatic α-cells	Lesser Extent	No	[73]
4	βTC1	Mice	Pancreatic β-cells	Yes	Yes	[73]
5	βTC3	Mice	Pancreatic β-cells	Yes	No	[26,117]
6	βTCtet	Mice	Pancreatic Islet β-cells	Yes	Yes	[73]
7	AML-12	Mice	Liver	Yes	Yes	Author *
8	AR42J	Rat	Pancreatic Exocrine	Lesser Extent	No	[73]
9	BaF3	Mice	B Lymphocytes	Yes	Very low	[18]
10	C2BBe1	Human	Clone of Caco-2	Yes	NA	[8,25,32]
11	C2C12	Mice	Muscle	Yes	Yes	Author *
12	Caco-2	Human	Colon	Low level	NA	Author *, [8,34]
13	Cardiomyocytes	Mice	Heart	Yes	Yes	[23]
14	CBS	Human	Colorectal	No	NA	[34]
15	CHO-K1	Hamster	Ovary	Yes	Yes	[54,55,118,120]
16	CMEC/D3	Human	Brain Endothelium	NA	Yes	[64]
17	CMT93	Mice	Rectal Cell	Yes	Yes	Author *
18	COS-7	Monkey	Kidney	Yes	Yes	[54]
19	FET	Human	Colon	No	NA	[30,34]
20	GLUTag	Mice	Intestinal Enteroendocrine Cells	Yes	Yes	Author *, [11,121]
21	hMADS	Human	Adipose tissue-derived stem cells	Yes	Yes	[15]
22	H9C2	Rat	Heart/Myocardium	Yes	Yes	Author *
23	HBMEC	Human	Primary Brain Microvascular Endothelial Cells	NA	Yes	[64]
24	HCT116	Human	Colon	No	Yes	[34,99]
25	HCT8	Human	Colon	No	NA	[34]
26	HEK293T	Human	Embryonic Kidney	Yes	Yes	[29,54,103,122]
27	HeLa	Human	Cervix	Yes	NA	[29]
28	Hepa1-6	Mice	Liver	Yes	Yes	Author *
29	HEPG2	Human	Liver	Yes	Yes	Authors *
30	HLE	Human	Liver cells	Yes	Yes	[123]
31	HRCEs	Human	Kidney tissues	Yes	Yes	[124]
32	HT-29	Human	Colon	Yes	NA	Author *, [8,34]
33	HuH-7	Human	Hepatocellular	Yes	Yes	[123]
34	HuTu-80	Human	Duodenum Epithelium	Yes	Yes	[125]
35	INS1	Rat	Pancreatic Islet	Yes	NA	[25,32]
36	JHH-4	Human	Hepatic cell	Yes	Yes	[123]
37	Jurkat T cells	Human	T-Lymphocyte Cells	NA	Yes	[41]
38	K562	Human	Bone Marrow	Very low	NA	[18]
39	L-10	Mice	Lymphoid Cells	Yes	No	[73]
40	Ltk	Mice	Adipose fibroblast cell	Yes	No	[34,73]
41	MCF7	Human	Mammary Gland	Yes	Yes	[29,65]
42	MDA-MB-231	Human	Breast Epithelial cells	Yes	Yes	[126]
43	MDA-MB-436	Human	Breast Epithelial cells	Yes	Yes	[34,126]
44	MEFs	Mice	Embryonic Fibroblast	Yes	No	[30]
45	Min6	Mice	Pancreatic Endocrinal Cells	Yes	Yes	[16,32,73,74,117,127]
46	NCI-H716	Human	Intestinal Endocrinal L-cell	Yes	Yes	[105,125]
47	NCM-640	Human	Colon Epithelial cells	Very Low	NA	[8]
48	Neuro2A	Mice	Brain	Barely	Barely	[23]
49	NIH-3T3	Mice	Embryonic Cells	No	No	[73]
50	Ob-Luc	Mice	Adipocytes	NA	Yes	[97]
51	Raw264.7	Mice	Macrophages	Yes	NA	Author *, [29]
52	SK-N-SH	Human	Brain	Yes	Yes	Author *
53	SW480	Human	Colon	No	NA	[29,34,54,103,122]
54	SW620	Human	Colon	No	NA	[34]
55	SW872	Human	Liposarcoma	Yes	No	[128]
56	T-84	Human	Colon Epithelial cells	Very Low	NA	[8]
57	THP-1	Human	Monocyte	Yes	Yes	Author *
58	U937	Human	Myeloid Lymphocytes	Yes	NA	[18,54,55,118,120]

* Authors- we have confirmed the expression in our lab by qRT-PCR.

**Table 2:** Physiological function of FFAR2 in humans and mice.

S. No.	Tissue/Organ	Research Findings	Ref.
**Human**			
1	Intestinal L-cells	-Secrete GLP-1 and PYY in response to glucose via FFAR2 signaling.	[3,11,76,162]
2	Primary Neutrophils	-Cmp1 and CATPB function as an agonist and antagonist for the neutrophil FFAR2 respectively. -Cmp1 and acetate activates the phospholipase C-inositol phosphate 3 (IP_3_) Ca^2+^ signaling while CATPB inhibits it. -Cmp1 act as a potent activator of the NADPH -oxidase in TNF-α-primed neutrophils with increased release of superoxide. -Moreover, Cmp1 triggered NADPH oxidase activity was inhibited by PTX.	[163]
3	Primary Monocytes	-Non-responders of Cmp1 shows no transient rise in intracellular Ca^+2^. -Human monocyte FFAR2 reduces inflammatory cytokine expression in response to acetate. -FFAR2 modulates p38-MAPK, Akt, and ERK signaling in response to acetate and FFAR2 agonist (CFMB).	[22,163,164]
4	Primary Lymphocytes	-Non-responders to Cmp1 with no transient rise in intracellular Ca^+2^.	[22,163]
5	Peripheral blood mononuclear cells (PBMCs)	-mRNA expression of FFAR2 upregulate in PBMCs in Type 1 Diabetes (T1D) patient via NFκB. -Overexpression induced cell apoptosis through ERK signaling. -Stimulated PBMCs for cytokine production in the presence of lipopolysaccharides (LPS) with and/or without acetate along with anti-FFAR2 antibody.	[29,42,163]
6	Primary Adipocytes	-FFAR2 expressed in the human multipotent adipose tissue-derived stem cells (hMADS). -SCFA acetate (luminal and systemic) are responsible for the antilipolytic response. -Treating with G_i_-sensitive PTX inhibitors prevents anti-lipolytic response develop by acetate. -A mixture SCFA reduces plasma FFA in DIO mice along with beige adipogenesis marker. -So, colonic or systemic acetate modulation helps in improving the insulin resistance in human adipocytes via FFAR2 mediated attenuation of HSL phosphorylation.	[15,19,29,42]
7	Colon	-Luminal propionate stimulates FFAR2 pathway through PYY mediation confirmed by Y1 and Y2 antagonist (BIBO3304 and BIIE0246). -FFAR2 signaling expressed evenly in the entire intestine mostly at colon in the presence of FFAR2 agonist PA.	[15,19,165]
**Mouse/Rodent**			
1	Pancreatic β-cells	-mRNA expression of FFAR2 upregulated through increase in pancreas β-cell expansion. -Increased β-cell contributes to more insulin secretion. -FFAR2 KO mice reduces gestational pancreatic β-cell expansion during pregnancy. -FFAR2 KO mice gestational glucose tolerance worsened even under antibiotic treatment and further deteriorated during second pregnancy. -Antibiotic modulation of gut microbiota does not disrupt the contribution of FFAR2 to gestational glucose tolerance. -FFAR2 acts as a novel target for β-cells adaptation to pregnancy-induced insulin resistance during to maintain normal glucose homeostasis. -FFAR2 a novel therapeutic target to stimulate β-cell growth and Proliferation.	[25,96,166,167]
2	Primary Pancreatic Islet	-SCFAs such as Acetate, propionate, and butyrate administration have no effect on insulin and glucagon secretion regardless of glucose level. -CFMB (FFAR2 agonist) has a significant effect in increasing the somatostatin and insulin secretion whereas no effect was observed in glucagon synthesis. -Mediate an inhibition of insulin secretion by coupling to Gi-type G Proteins -Under type 2 diabetic condition acetate concentration increases in pancreatic islet and systemic circulation -FFAR2 antagonist might increase insulin secretion in type 2 diabetes -Double knock-out of FFAR2 and FFAR3 altered the glucose tolerance in diabetic condition.	[28,168]
3	Ileum	-Bacterial metabolites, propionate, activate ileal mucosal FFAR2 to decrease hepatic glucose production. -Propionate stimulate GLP-1r dependent neuronal network to regulate glucose production activated through ileal FFAR2 signaling. -Regulate glucose homeostasis.	[28,169]
4	Macrophages	-Inducing apoptosis of infiltrated macrophages to pancrease through upregulation of FFAR2. -Improved glucose homeostasis in diabetic mice by treating with FFAR2 agonist, acetate and phenylacetamide 1.	[29,169]
5	Peripheral blood mononuclear cells (PBMCs)	-Dextran sodium Sulphate (DSS) -induced colon shortening, mucosal thickness, inflammatory cell infiltration, and crypt damage were ameliorated by acetate treatment in C57BL/6 mice. -Stimulated PBMCs in FFAR2 KO mice for cytokine production in the presence of lipopolysaccharides (LPS) with and/or without acetate. -DSS-induced colitis is exaberated in FFAR2 KO mice through increase in pro-inflammatory cytokines such as TNF-α and IL-17 with decrease of anti-inflammatory cytokine IL-10 in the colonic mucusa.	[29,42]
6	Neutrophil	-FFAR2 recognizes propionate and butyrate and expressed abundantly in polymorphonuclear (PMN) leukocytes. -FFAR2 mediated SCFA-induced chemotaxis through p38 MAPK signaling pathway. -Inhibiting FFAR2 mediated signaling a promising way for inhibiting the migration of PMN at the site of intestinal inflammation. -Under influenza infection, in FFAR2 KO along with wild type mice showed decrease neutrophil infiltration to airway.	[43,114]
7	Immune Cells	-FFAR2 KO mice develops unresolving or exaberated inflammation in colitis, arthritis and asthma mice model. -FFAR2 KO mice shows inflammatory action related to increase in the production of inflammatory mediators by increased in immune cell recruitment. -Germ-free mice, which are devoid of bacteria and express little or no SCFAs, showed similar dysregulation of certain inflammatory response. -SCFA-FFAR2 interaction has profound effect on normal resolution of certain inflammatory response with a molecular link between diet, gastrointestinal bacterial metabolism and immune response.	[35,40,43]
8	Monocytes	-Mice monocyte showed increased in IL-1α and IL-1β cytokine expression in response to acetate. -Even in FFAR2/3 KO mouse monocyte display elevate cytokine response on treatment with SCFAs. -SCFA does not act through FFAR2 to modulate mice monocyte inflammatory responses.	[22,35]
9	L-cells	-GLP-1 synthesis was enhanced in the presence of phosphodiesterase inhibitor isobutyl methyl xanthine (IBMX). -FFAR2 expression in small intestine and colonic L-cells as compare to non-L-cell population. -Induces GLP-1 and PYY secretion via glucose dependent mechanism. -SCFAs triggered Ca^2+^ elevation in L-cells with enhanced GLP-1 and PYY secretion through G_q_-mediated pathway, implicating FFAR2 signaling involvement. -Synthetic phenylacetamide agonist of FFAR2, CFMB, mobilizes more intracellular Ca^2+^ in L-cells and elevates GLP-1 hormone secretion, in the presence of DPPIVi but not in its absence in mice.	[11,22,162,170]
10	Colonic Mucosa	-FFAR2 express in the colonic mucosa -Withdrawal of ceftriaxone antibiotic leads to reduction in SCFA concentration and increase in increased number of conditionally pathogenic *Enterobacteria*, *E. coli*, *Clostridium*, *Staphylococcus spp*., and hemolytic bacteria in colonic gut. -FFAR2 immune regulation mechanism get hamper with increase in cytokine concentration in colonic mucosa. -Increase histopathology condition of colitis with goblet cell dysfunction, colonic dilatation and wall thickening, ultimate leads to IBD.	[78]
11	Enterochromaffin cells	-FFAR2 agonist PA1 (Phenylacetamide 1) in a dose-dependent manner stimulate HCO_3_^-^ secretion, even prior exposed to DPPIV inhibitor NVP728. -HCO_3_^-^ secretion stimulate by activated FFAR2 through muscarinic and 5-HT_4_ receptor signaling rather than through VIP, CCK and GLP-2 pathway. -Moreover, SCFAs (mostly acetate) activate FFAR2 and FFAR3 followed by 5-HT and GLP-2 release.	[171]
12	Mast cell	-Rat intestinal lamina propria mast cells expressed FFAR2 along with 5-hydroxytryptophan (5-HT). -The activated mucosal FFAR2 act on the nearby nerve endings at 5-HT_3_ serotogenic receptors. -SCFAs stimulate PYY and 5-HT secretion from ileum and colonic endocrine cells by activating FFAR2 receptor.	[76,171]
13	Stomach	-The villi and microvilli of gastric brush cells reveal expression of FFAR2 (at gene and protein level) in the mice stomach.	[7,76,172]
14	Lungs	-Expressed in the mice lungs. -SCFAs modulate allergy airway inflammation in mice lungs via FFAR2 signaling.	[6,7,152,172]
15	Muscle	-Expressed FFAR2 in smooth muscle cells of small resistance vessels. -SCFAs produced from gut microbiota modulate the blood glucose level.	[6,20]

FFAR2: Free fatty acid receptor 2; GLP-1: Glucagon-like peptide 1; PYY: Peptide YY; Cmp1: Compound 1 (3-benzyl-4-(cyclopropyl-(4-(2,5-dichlorophenyl)thiazol-2-yl)amino)-4-oxobutanoic acid; ERK: Extracellular signal-regulated kinase); CATPB: (S)-3-(2-(3-chlorophenyl)acetamido)-4-(4-(trifluoromethyl)phenyl)butanoic acid; GLPG0974: 4-[[(2R)-1-(1-benzothiophene-3-carbonyl)-2-methylazetidine-2-carbonyl]-[(3-chlorophenyl)methyl]amino]butanoic acid; IP_3_: Inositol phosphate 3; NADPH: Nicotinamide adenine dinucleotide phosphate; TNFα: Tumor Necrosis Factor alpha; PTX: Pertussis toxin; MAPK: Mitogen-activated protein kinase; Akt: Protein Kinase B; ERK: Extracellular signal-regulated kinase; CFMB: [( S)-2-(4-chlorophenyl)-3,3-dimethyl- N-(5-phenylthiazol-2-yl)butamide; PBMCs: Peripheral blood mononuclear cells; T1D: Type-1 Diabetes; LPS: Lipopolysaccharides; hMADS: human multipotent adipose tissue-derived stem cells; SCFA: Short chain fatty acids; FFA: Free fatty acids; DIO: Diet induced obesity; HSL: Hormone-sensitive lipase; FFAR3: Free fatty acid receptor 3; GLP-1r: Glucagon-like peptide 1 receptor; DSS: Dextran sodium Sulphate; KO: Knock out; LPS: Lipopolysaccharides; IL: Immunoglobulin; PMN: Polymorphonuclear; SCFA: Short chain fatty acid; IBMX: Isobutyl methyl xanthine; DPPIVi: Dipeptidyl peptidase 4 inhibitor; PA1: Phenylacetamide 1; ACh: Acetylcholine; 5-HT: 5-Hydroxytryptophan; 5-HT_3_: 5-Hydroxytryptophan type 3; HCO_3_^-^: Bicarbonate: VIP: Vasoactive intestinal peptide; CCK: Cholecystokinin; GIP: Glucose-dependent insulinotropic peptide; IGN: Intestinal Gluconeogenesis.

## Abbreviations

SCFAs	Short-chain fatty acids
FFAR2	Free fatty acid receptor 2
FFAR3	Free fatty acid receptor 3
GPCRs	G-coupled protein receptors
T1D	Type 1 diabetes
T2D	Type 2 diabetes
IBD	Inflammatory bowel disease
TM	Transmembrane
PBMCs	Peripheral blood mononuclear cells
PMNs	Polymorphonuclear cells
DCs	Dendritic cells
WAT	White adipose tissue
BAT	Brown adipose tissue
bMEC	Bovine mammary epithelial cell line
SNS	Sympathetic nervous system
CNS	Central nervous system
CRC	Colorectal cancer
SCG	Superior cervical ganglia
CSMG	Celiac sympathetic-mesenteric ganglia
GI	Gastrointestinal
AAs	Amino acids
Tyr	Tyrosine
Ile	Isoleucine
Arg	Arginine
Glu	Glutamate
EL	Extracellular loop
SCAs	Small carboxylic acids
Trp	Tryptophan
Gln	Glutamine
His	Histidine
Thr	Threonine
Phe	Phenylalanine
Leu	Leucine
SAR	Structure activity relationship
Å	Angstrom
SASA	Solvent accessible surface area
H	Hydrogen
PYY	Peptide YY
Cmp1	Compound 1
CFMB/Phenylacetamide 1	[(S)-2-(4-chlorophenyl)-3,3-dimethyl- N-(5-phenylthiazol-2-yl)butamide
AMG-7703	(2S)-2-(4-chlorophenyl)-3-methyl-N-(1,3-thiazol-2-yl)butanamide
Val	Valine
4-CMTB	2-(4-chlorophenyl)-3-methyl-N-(thiazole-2-yl)butanamide
2CTAP	4-((4-(2-chlorophenyl)thiazole-2-yl)amino)-4oxo-3-phenylbutanoic acid
BTI-A-404	[4-[4-(dimethylamino)phenyl]-N-(3,5-dimethylphenyl)-6-methyl-2-oxo-1,2,3,4-tetrahydro-5-pyrimidinecarboxamide]
BTI-A-292	[4-[4-(dimethylamino) phenyl]-N-(4,5-dimethylphenyl)-6-methyl-2-oxo-1,2,3,4-tetrahydro-5-pyrimidinecarboxamide]
CATPB	(S)-3-(2-(3-chlorophenyl)acetamido)-4-(4-(trifluoromethyl)phenyl)butanoic acid
GLPG0974	4-[[(2R)-1-(1-benzothiophene-3-carbonyl)-2-methylazetidine-2-carbonyl]-[(3-chlorophenyl)methyl]amino]butanoic acid
Compound 1	3-benzyl-4-(cyclopropyl-(4-(2,5-dichlorophenyl)thiazol-2-yl)amino)-4-oxobutanoic acid
Phenylacetamide 2/Compound 44	(S)-2-(4-chlorophenyl)-N-(5-fluorothiazol-2-yl)-3-methylbutanamide
Phenylacetamide 58	(S)-2-(4-chlorophenyl)-3,3-dimethyl-N-(5-phenylthiazol-2-yl)butanamide
1-MCPC	1-methylcyclopropane carboxylate; AR420626: N-(2,5-dichlorophenyl)-4-(furan-2-yl)-2-methyl-5-oxo-1,4,5,6,7,8-hexahydro-quinoline-3-carboxamide
CF_3_-MQC	N-(2-methylphenyl)-4-[5-(2-trifluoromethoxy-phenyl)-furan-2-yl)-2-methyl-5-oxo-1,4,5,6,7,8-hexahydroquinoline-3-carboxamide
MQC	N-[2-methylphenyl]-[4-furan-3-yl]-2-methyl-5-oxo-1,4,5,6,7,8-hexahydroquinoline-3-carboxamide
DSS	Dextran sodium sulphate
LPS	Lipopolysaccharide
MAPK	Mitogen-activated protein kinase
Ig	Immunoglobulin
CRAMP	Cathelicidin related antimicrobial peptide
NFκB	Nuclear factor kappa-light-chain-enhancer of activated B cells
AMPK-α	5’ adenosine monophosphate-activated protein kinase
PI3K	Phosphatidylinositol 3-kinase
Akt	Protein Kinase B
ERK	Extracellular signal-regulated kinases
INFγ	Interferon gamma
PTX	Pertussis toxin
JNK	c-Jun N-terminal kinase
iNOS	Induced nitric oxide synthase
HUVEC	Human umbilical vein endothelial cells
HDACi	Histone deacetylases inhibitor
Treg	Regulatory T-cells
WT	Wild type
mTOR	The mammalian target of rapamycin
eNOS	Exhaled nitric oxide
TNF-α	Tumor Necrosis Factor alpha
GIT	Gastrointestinal tract
GLP-1	Glucagon-like peptide 1
POMC	Pro-opiomelanocortin
NPY	Neuropeptide Y
AgRP	Agouti-related peptide
qRTPCR	Quantitative reverse transcription polymerase chain reaction
5-HT	Hydroxytryptamine
5-HCO_3_^-^	Bicarbonate
cAMP	Cyclic adenosine monophosphate
FACS	Fluorescence-activated cell sorting
mRFP	Monomeric red fluorescent protein
GIP	Gastric inhibitory polypeptide
RYGB	Roux-en-Y gastric bypass
M-cells	Myeloid cells
IECs	Intestinal epithelial cells
5-HT	5-Hydroxytryptamine
IL	Interleukin
Tjp1	Tight junction protein 1
Ocln	Occludin
Cldn	Claudin
Muc	Mucin
AMPK	AMP-activated protein kinase
HIF	Hypoxia-inducible factor
DSS	Dextran sodium sulphate
TNBS	Trinitrobenzoic sulphonic acid
PNS	Peripheral nervous system
BBB	Blood brain barrier
TH	Tyrosine hydroxylase
PLC	Phospholipase C
nAChR	Nicotinic acetylcholine receptor
Ca	Calcium
IP	Intraperitoneal
DIO	Diet induced obese
HFD	High fat diet
siRNA	Small interfering ribonucleic acid
PPARγ	Peroxisome proliferator-activated receptor gamma
hMADS	Human multipotent adipose tissue-derived stem
NC	Normal chow
OGTT	Oral glucose tolerance test
IM-BAT	Immortalized brown adipocyte cell line
GSIS	Glucose-stimulated insulin secretion
DSS	Dextran sodium sulphate
DAI	Daily activity index
